# Imaging of Skull Base Tumors

**DOI:** 10.3390/tomography9040097

**Published:** 2023-06-21

**Authors:** Bilal Battal, Carlos Zamora

**Affiliations:** Division of Neuroradiology, Department of Radiology, University of North Carolina School of Medicine, Chapel Hill, NC 27599, USA; carlos_zamora@med.unc.edu

**Keywords:** neuroimaging, skull base, neoplasms, clivus, cranial fossa, temporal bone

## Abstract

The skull base provides a platform for supporting the brain while serving as a conduit for major neurovascular structures. In addition to malignant lesions originating in the skull base, there are many benign entities and developmental variants that may simulate disease. Therefore, a basic understanding of the relevant embryology is essential. Lesions centered in the skull base can extend to the adjacent intracranial and extracranial compartments; conversely, the skull base can be secondarily involved by primary extracranial and intracranial disease. CT and MRI are the mainstay imaging methods and are complementary in the evaluation of skull base lesions. Advances in cross-sectional imaging have been crucial in the management of patients with skull base pathology, as this represents a complex anatomical area that is hidden from direct clinical exam. Furthermore, the clinician must rely on imaging studies for therapy planning and to monitor treatment response. It is crucial to have a thorough understanding of skull base anatomy and its various pathologies, as well as to recognize the appearance of treatment-related changes. In this review, we aim to describe skull base tumors and tumor-like lesions in an anatomical compartmental approach and present imaging methods that aid in diagnosis, management, and follow-up.

## 1. Introduction

Skull base lesions pose a significant diagnostic challenge as patients frequently present with limited and nonspecific clinical signs and symptoms. Imaging is therefore critical in the detection and differential diagnosis and can characterize lesions according to the site of origin, growth pattern, and tissue characteristics. In particular, magnetic resonance imaging (MRI), with conventional and advanced sequences, is a robust tool that can accurately define the lesion extent, which is essential for selecting optimal treatment strategies, anticipating surgical complications, and delineating targets for radiation therapy [1,2,3]. Both computed tomography (CT) and MRI have distinct advantages and disadvantages and are often complementary. The purpose of this review is to discuss the differential diagnosis of common skull base tumors and tumor-like lesions and their characteristic imaging findings in an anatomical compartmental approach and present imaging methods that aid in diagnosis, management, and follow-up.

## 2. Anatomic Considerations

The skull base forms the floor of the cranial cavity and separates the brain from other facial and neck structures. It is subdivided into three regions: the anterior, middle, and posterior cranial fossae, comprising paired (frontal, temporal) and unpaired (ethmoid, sphenoid, occipital) bones. The petro-occipital fissure subdivides the middle cranial fossa into one central and two lateral compartments. The central skull base is separated from the anterior skull base by the posterior border of the lesser and greater sphenoid wings and planum sphenoidale. The dorsum sellae, posterior clinoid processes, and posterior petrous ridges comprise the separation between the central and posterior skull base [1,2,3,4,5]. The skull base is perforated by numerous foramina, which allow the passage of vessels and nerves and provide communication between the intracranial and extracranial compartments. These are important sites to scrutinize on the imaging evaluation of the skull base, as the cranial nerves can also be a source of pathology and represent an important pathway for disease extension [2,3].

## 3. Imaging Techniques

Given the complex anatomy of the skull base and the small size of its neurovascular structures, it is crucial that imaging be performed using thin slices and provide multiplanar reconstructions. CT is the modality of choice for the delineation of fine bony detail, detection of matrix calcification, and identification of aggressive features, such as bone destruction and erosion. CT should be performed using a slice thickness of 0.5–1 mm with a bone algorithm reconstruction, including multiplanar reformats [1].

High-resolution MRI should be performed at a minimum of 1.5 Tesla and ideally at 3.0 Tesla, as the latter provides a higher signal-to-noise ratio, which is important to resolve small structures, provide lesion detail, and identify perineural disease [1,6]. Routine skull base MRI protocols should include T1, T2, and postcontrast T1-weighted imaging (T1WI) at a slice thickness of ≤3 mm. Isovolumetric (3D) postcontrast T1WI is the most sensitive technique for demonstrating perineural tumor spread, whose presence is associated with a worse prognosis and is an important consideration for treatment planning [1,7,8,9]. For optimal resolution, we perform these at a ≤0.8 mm slice thickness, utilizing a small field of view. Fat-suppression techniques are crucial in T2WI and postcontrast T1WI to accurately assess tumor extent and perineural spread by suppressing bright background signals from fat [1,10]. Significant progress has been made in the imaging of skull base tumors, particularly through the use of high-resolution balanced steady-state free precession MRI techniques (such as CISS (constructive interference in steady state)), refinements in advanced angiographic techniques such as MR vessel wall imaging, and advancements in functional imaging methods, including diffusion-weighted imaging (DWI) and positron emission tomography (PET). These developments have greatly improved the ability to accurately detect, characterize, and precisely localize skull base tumors, enhancing diagnostic accuracy and treatment planning.

High-resolution isotropic CISS, or its analog FIESTA-C, is necessary for the accurate visualization of the cisternal portions of the cranial nerves (CNs), owing to the intrinsic bright signal from the surrounding cerebrospinal fluid (CSF) [1,6,7]. While these are primarily heavily T2-weighted techniques, they also exhibit T1 effects, allowing them to demonstrate contrast enhancement. Administration of intravenous contrast material in CISS or FIESTA-C has recently gained more attention for skull base imaging and is essential for visualizing the interdural CN segments and depicting their relationship with skull base lesions [11,12].

DWI is valuable for demonstrating purulent material (e.g., in skull base osteomyelitis) or neoplastic lesions that are highly cellular. However, one drawback of traditional EPI-based DWI techniques in the skull base is the occurrence of geometric distortion due to magnetic field inhomogeneities at brain–bone–air interfaces, as well as susceptibility artifact. Recent advances include the development of non-EPI techniques as well as readout segmented EPI, which can mitigate these artifacts but result in increased scanning times [13].

Bone marrow invasion may be evident on unenhanced T1WI or STIR (short tau inversion recovery) as an abnormal signal replacing the normal bone marrow fat. Advanced MRI techniques, including susceptibility-weighted imaging, perfusion-weighted imaging, and MR spectroscopy, can also aid in improving diagnostic accuracy, although their application in the skull base region can be challenging.

CTA and MRA techniques provide information about the patency of the intracranial arteries and their relation to tumors, which is important for surgical planning. High-resolution MR vessel wall imaging is useful to characterize associated vasculitis due to its ability to demonstrate enhancement in the area of the vessel wall inflammation.

Digital subtraction angiography (DSA) is the gold standard for delineating the arterial supply of hypervascular tumors, particularly for lesions amenable to embolization. This may be performed as an adjunct to minimize bleeding before radical surgery or as a palliative measure when curative resection is not possible [1,14].

^18^F-fluoro-deoxyglucose PET or PET-CT is commonly used to assess the metabolic activity of skull base lesions, locate primary tumors, or identify additional lesions in other areas that may affect cancer staging [5].

## 4. Skull Base Tumors

### 4.1. Tumors Occurring in All Regions of the Skull Base

#### 4.1.1. Meningioma

Meningiomas are extra-axial dural-based tumors arising from arachnoid cap cells. While most of them are benign (WHO grade 1), they can be atypical (grade 2) and rarely malignant (grade 3). Meningiomas usually appear isodense to gray matter on noncontrast CT, and isointense to slightly hyper-intense to gray matter on MRI, with avid contrast enhancement, although there are multiple histological subtypes and imaging appearance can vary. Calcifications are present in 20–30% [15]. Meningiomas have been classically associated with a dural tail reflecting reactive thickening and perilesional meningeal enhancement, which is seen in 60–72% of tumors. However, this finding is not entirely specific, is less commonly seen in the skull base, and may be present in other processes such as lymphoma, tuberculosis, sarcoid, immunoglobulin G4-related disease, granulomatosis with polyangiitis, and fungal infection [16,17].

**Figure 1:**
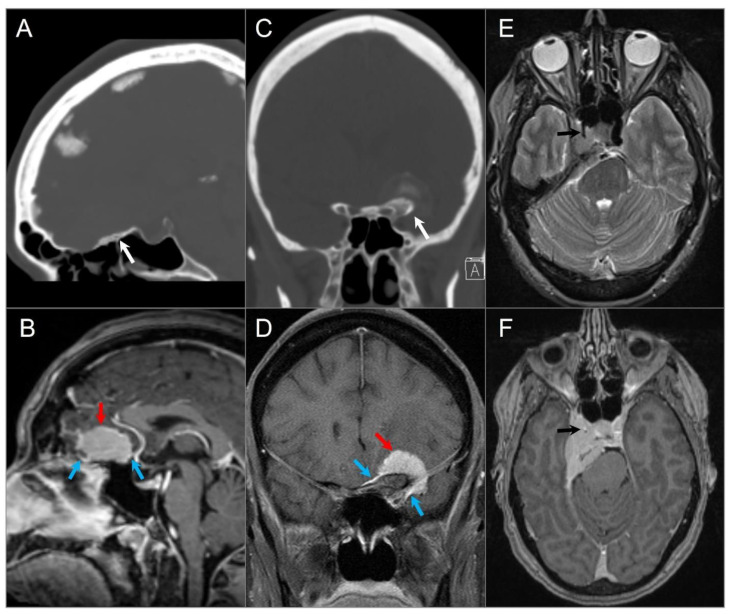
Meningiomas in three different patients. (**A**) Sagittal CT and (**B**) sagittal postcontrast T1W MR images demonstrate a homogenously enhancing extra-axial mass lesion (red arrow) at the anterior cranial fossa/planum sphenoidale with small dural tails (blue arrows) and hyperostosis (white arrow). (**C**) Coronal CT and (**D**) coronal postcontrast fat-saturated T1W images show an anterior clinoid process meningioma (red arrow) with dural tails (blue arrows), hyperostosis (white arrow), and tumoral calcification. (**E**) Axial T2W and (**F**) axial postcontrast T1W images reveal a right cavernous sinus meningioma extending through the petroclival dura, tentorium, and paraclinoid dura. The mass completely encases the right ICA with significant narrowing (black arrows).

**Figure 2:**
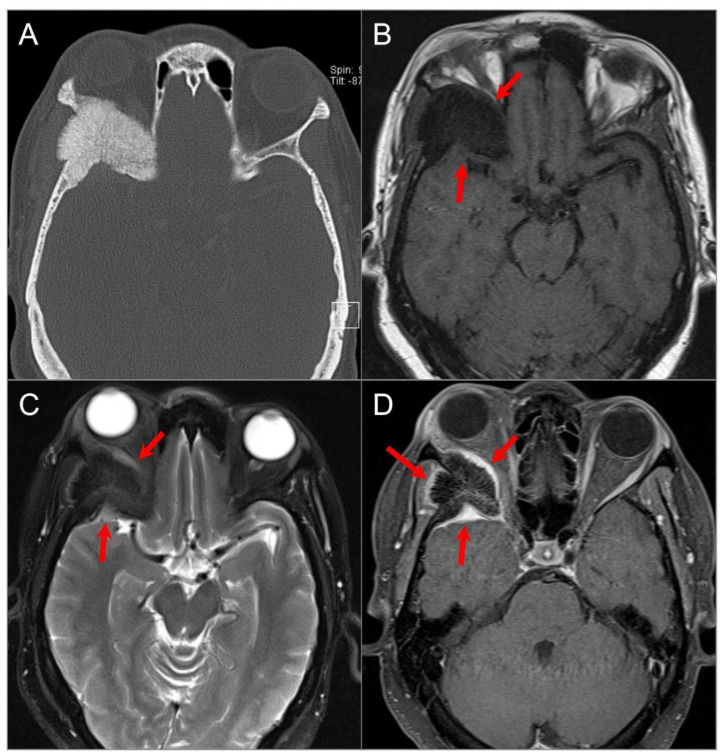
Intraosseous meningioma. (**A**) Axial CT shows a marked osseous expansion of the right posterolateral orbital wall and a greater wing of the sphenoid with a spiculated appearance. (**B**) Axial T1W and (**C**) STIR T2W images show marked hypointensity in the lesion with thin surrounding soft tissue components along the lateral orbital wall and temporal fossa (arrows). (**D**) Axial fat-suppressed postcontrast T1W image demonstrates heterogenous transosseous enhancement and avid contrast enhancement in the soft tissue components of the lesion along the temporal dura and orbital/temporal periosteum (red arrows). There is associated displacement of extraocular muscles and proptosis.

Meningiomas that abut the base of the skull are associated with focal hyperostosis, possibly secondary to osteoblastic stimulating factors, tumoral invasion, or a combination of both, although the definite pathogenesis is not fully understood [2]. The enlargement of adjacent paranasal sinuses has also been associated with anterior cranial fossa meningiomas, particularly along the planum sphenoidale, where there may be an upward blistering of the sphenoid sinus (pneumosinus dilatans). Petroclival and parasellar meningiomas tend to involve the cavernous sinus, optic canal, and Meckel’s cave. The narrowing of encased arteries is typically seen in meningiomas and may be a useful sign to distinguish them from pituitary adenomas, which do not result in arterial narrowing (Figure 1) [6]. En plaque meningiomas are characterized by sheetlike dural thickening rather than a globular tumor growth and more prominent subjacent hyperostosis. The distinction between en plaque meningiomas with a prominent osseous component and intraosseous meningiomas is rather vague and relatively arbitrary (Figure 2). Higher-grade meningiomas may demonstrate lytic and destructive features; however, alternative pathologies such as solitary fibrous tumor or metastasis should also be considered in such cases. MR spectroscopy does not play a routine role in diagnoses, but short TE proton (^1^H) MR spectroscopy may show an alanine peak at 1.3–1.5 ppm and increased glutamine and glutamate peaks, which may be useful in equivocal cases [2].

#### 4.1.2. Schwannoma

Schwannomas are benign, slow-growing neoplasms that arise from Schwann cells and can involve CNs III–XII in the central and posterior skull base. Although olfactory nerves lack Schwann cells and are thus typically spared, schwannomas arising from the olfactory filia directly below the cribriform plate have been reported but are extremely rare [18]. The optic nerves (CN II) are not true cranial nerves, but rather an extension of white matter tracts and are therefore not associated with schwannomas.

Schwannomas account for 8.5% of all intracranial tumors and are usually seen in adulthood [6]. Most intracranial schwannomas originate from CN VIII, primarily involving the cerebellopontine angle (CPA) cistern (90%), followed by CN V (1–8%) and CN VII [19]. Intraorbital schwannomas commonly arise from supraorbital and supratrochlear nerves in the upper orbital cavity. In the central para-sellar skull base, CN V schwannomas predominate, while CN III, IV, and VI schwannomas are rare, except in the setting of NF2-related schwannomatosis [19]. NF2-related schwannomatosis is a phakomatosis that also increases the chances of multiple inherited meningiomas and ependymomas. Vestibular schwannomas are the most frequent form of posterior skull base schwannomas, accounting for 85% of all CPA masses and 6–10% of all intracranial tumors [20,21]. Schwannomas of CN X and especially of CNs IX, XI, and XII are rare. The majority of schwannomas are slow-growing with an estimated progression rate of 1–2 mm/year [22].

**Figure 3:**
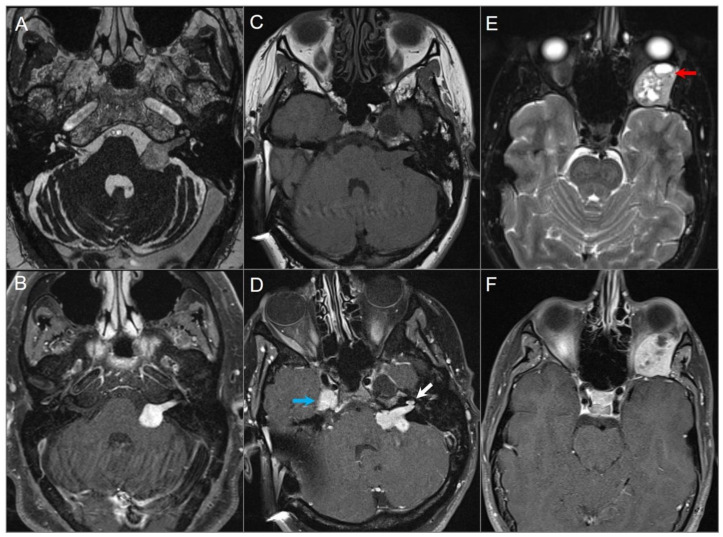
Schwannomas in three different patients. (**A**) Axial CISS and (**B**) axial postcontrast fat-suppressed T1W images show a well-defined, homogenously enhancing left CPA mass with extension into the left internal auditory canal. (**C**) Axial T1W and (**D**) postcontrast axial fat-suppressed T1W images show multiple schwannomas centered in the right Meckel’s cave (blue arrow) and the left CPA in a patients with NF2-related schwannomatosis. The left CPA mass completely fills the IAC and extends into the cochlea (white arrow). (**E**) Axial T2W and (**F**) axial postcontrast fat-suppressed T1W images reveal a left orbital extraconal, well-defined, heterogeneously enhancing intra-orbital schwannoma with bone remodeling/dehiscence and mass effect on the optic nerve and extraocular muscles. The mass contains multiple non enhancing areas of cystic degeneration with fluid–fluid levels likely due to hemorrhage (red arrow).

On MRI, schwannomas are well-circumscribed, round or lobulated, and generally solid T1-iso-, T2-hyperintense masses, which demonstrate avid contrast enhancement. A classic “ice cream on cone” appearance is typical for vestibular schwannomas involving the CPA, but small lesions may be purely intracanalicular. Schwannomas may demonstrate varying degrees of heterogeneity due to cystic change or, rarely, hemorrhage or calcification (Figure 3) [23,24,25]. Melanotic schwannomas are rare and exhibit intrinsic T1 hyperintensity. This can lead to confusion with lipomas, but using fat-saturated MRI sequences can aid in distinguishing between the two [26,27].

#### 4.1.3. Metastasis

**Figure 4:**
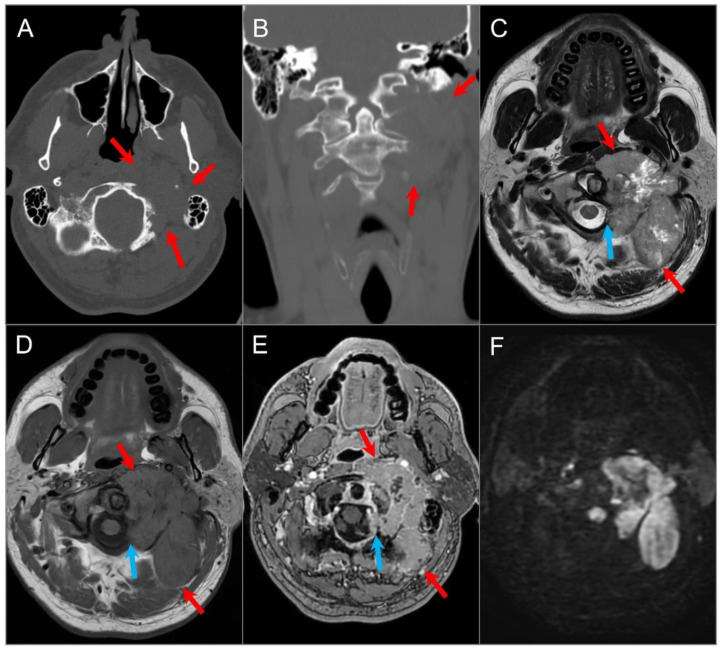
Skull base metastasis from RCC. (**A**) Axial and (**B**) coronal CT images show a lucent destructive soft tissue density mass lesion (red arrows) involving the left occipital bone, including occipital condyle, left lateral mass of C1, and inferomedial mastoid. (**C**) T2W, (**D**) noncontrast T1W, and (**E**) postcontrast T1W images reveal a bulky mass (red arrows) involving the left skull base and extending to the left retropharyngeal region and left epidural space (blue arrows). The lesion shows avid enhancement with central cystic/necrotic degeneration. (**F**) DWI image shows hyperintense signal in the solid components of the lesion consistent with restricted diffusion due to high cellularity.

Skull base metastases occur in approximately 4% of cancer patients and generally in those with advanced disease. They are most frequently found in the clivus, petrous apex, and sphenoid bone because of their higher marrow content. Although various tumors may give rise to skull base metastases, the most common primary tumors are breast, prostate, lung carcinoma, and lymphoma metastasis [6,28]. Hematogenous spread is the most common cause of metastases, but seeding through the valveless Batson venous plexus, the direct extension of sinonasal, nasopharyngeal or sphenoid tumors, and perineural spread commonly along branches of CN V may also occur. Skull base metastases are often clinically silent until there is CN involvement, which can lead to specific neuropathies [6]. Of these, CN VI palsy is the most frequently observed. The imaging appearance can be variable depending on tumor histology and lesions can have a lytic or sclerotic appearance on CT. On MRI, most lesions show T1 hypointense signals due to the replacement of normally hyperintense fatty marrow as well as contrast enhancement. T2 signal changes and contrast enhancement are best appreciated on fat-saturated sequences. Additionally, DWI sequences can be helpful to demonstrate bony metastases as they are frequently associated with restricted diffusion (Figure 4).

#### 4.1.4. Lymphoma

While primary lymphoma of the osseous skull base is exceedingly rare, direct invasion or perineural spread of sinonasal and neck lymphomas to the skull base is frequently seen. Intracranial lymphoma can manifest as various subtypes, including primary central nervous system (CNS) B-cell non-Hodgkin lymphoma (B-cell PCNSL), intravascular lymphomatosis (a rare form of extranodal non-Hodgkin lymphoma), T-cell PCNSL, and Hodgkin’s lymphoma [29,30]. Non-Hodgkin’s lymphomas may affect any site of the skull base, but involvement of the clivus and parasellar region is common and accounts for approximately 5% of head and neck malignancies [31]. In addition to non-specific systemic manifestations such as fever and weight loss, patients may experience an abrupt onset of localized symptoms such as nasal obstruction, epistaxis, headaches, and cranial neuropathies. A rapid improvement of symptoms with corticosteroid treatment is also a characteristic feature.

**Figure 5:**
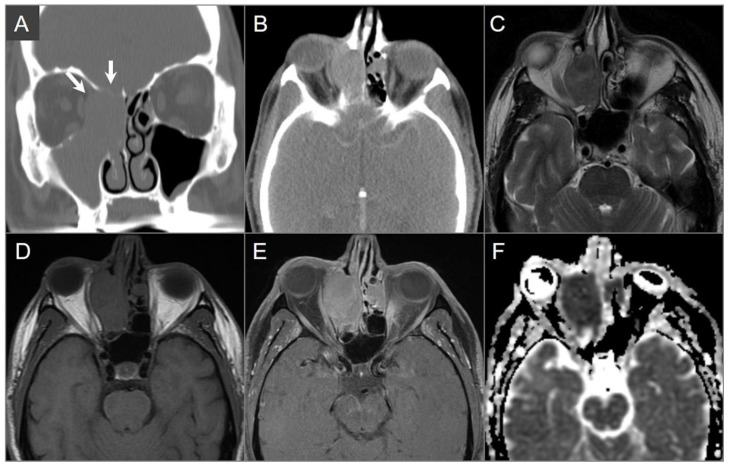
Diffuse large B cell lymphoma. (**A**) Coronal and (**B**) axial postcontrast CT images show an isodense soft tissue density-enhancing lesion, involving the right maxillary and ethmoid sinuses and superior nasal cavity with right medial orbital wall and right anterior skull base dehiscence (white arrows). (**C**) T2W, (**D**) noncontrast T1W, and (**E**) postcontrast fat-suppressed T1W images reveal homogenous enhancement of the lesion, which demonstrates isointensity on T1W and hypointensity on T2W images consistent with high cellularity. The lesion extends into the right extraconal orbital compartment and mildly compresses the right medial rectus muscle with a small component of the lesion in the left superior nasal cavity. (**F**) ADC map image shows a hypointense signal in the lesion consistent with restricted diffusion due to high cellularity.

**Figure 6:**
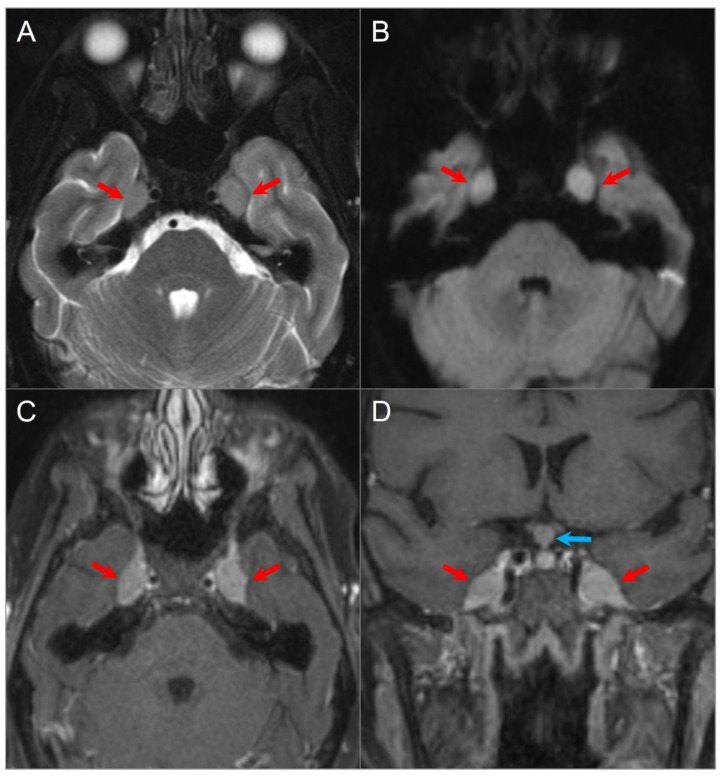
Meckel’s cave and pituitary stalk involvement of acute lymphoblastic leukemia. (**A**) Axial STIR T2W image shows well-defined nodular lesions in the bilateral Meckel’s caves (red arrows) with isointense signal relative to the cerebral cortex. (**B**) DWI image demonstrates mild restricted diffusion in the lesions. (**C**) Axial and (**D**) coronal fat-suppressed postcontrast images reveal homogeneous enhancement of the lesions. There is a nodular lesion in the pituitary stalk with similar signal characteristics (blue arrow).

On MRI, skull base lymphoma demonstrates intermediate signal intensity on all sequences, although T2 hyperintensity is also recognized [32]. Diffusion restriction with low ADC values between 0.51–0.59 × 10^−3^ mm^2^/s due to tumor hypercellularity is a helpful diagnostic clue that can be also used to assess follow-up and treatment response (Figure 5 and Figure 6). Bone destruction and excessive soft tissue mass are more prominent in T-cell lymphomas, while B-cell lymphomas usually present with a large soft tissue mass and associated bone remodeling [32,33].

#### 4.1.5. Extension of Head and Neck Malignancy

**Figure 7:**
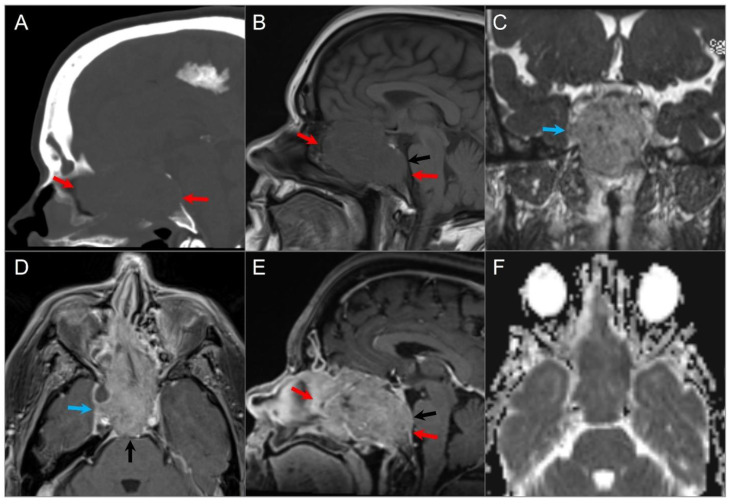
Skull base invasion of squamous cell Ca. (**A**) Sagittal CT image shows a hypodense soft tissue mass lesion centered in the superior nasal cavity, ethmoid, and sphenoid sinuses, which erodes the anterior skull base, sellar floor, and clivus (red arrows). (**B**) Sagittal noncontrast T1W, (**C**) coronal postcontrast CISS, (**D**) axial and (**E**) sagittal postcontrast T1W images demonstrate significant heterogeneous enhancement of the lesion with extension into the right cavernous sinus (blue arrow), prepontine cistern (black arrows), and anterior cranial fossa. (**F**) ADC map image shows mild restricted diffusion in the lesion, suggesting high cellularity.

**Figure 8:**
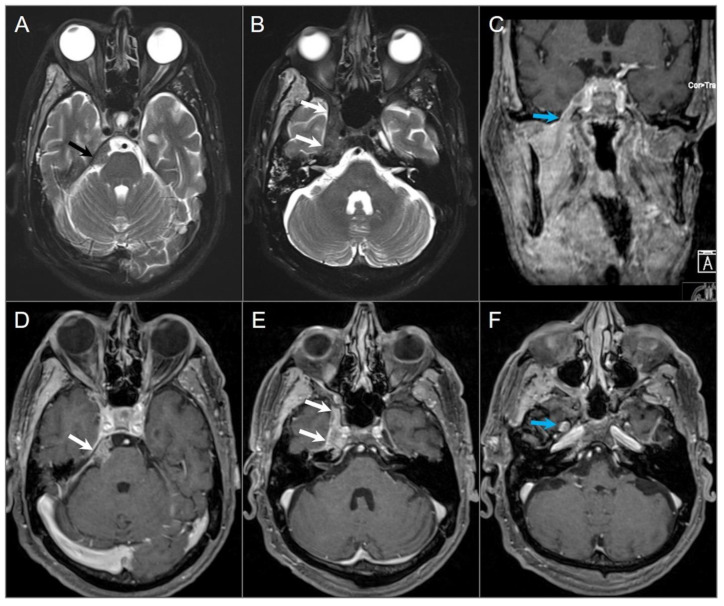
Indirect perineural invasion of the skull base by a previously surgically resected parotid adenoid cystic carcinoma. (**A**,**B**) Axial T2W image shows asymmetric infiltration of the inferior aspect of the right cavernous sinus, Meckel’s cave, and inferior orbital fissure by a hypointense soft tissue lesion (white arrows) extending into the right prepontine cistern along the trigeminal nerve cisternal segment (black arrows). (**C**) Coronal postcontrast T1W image demonstrates infiltrative heterogeneous enhancement in the right masticator and parapharyngeal spaces and a thickened enhancing right mandibular nerve (blue arrow), which provides a pathway for the perineural intracranial spread of the tumor. (**D**–**F**) Axial postcontrast T1W images show heterogenous enhancement of the perineural tumoral infiltration in the foramen ovale (blue arrow), inferior orbital fissure, Meckel’s cave, and right trigeminal nerve cisternal segment (white arrows).

Head and neck tumors may invade the skull base by either direct invasion or perineural spread (Figure 7). The latter can occur commonly in adenoid cystic carcinoma, squamous cell carcinoma, lymphoma, and melanoma [34], and typically along the branches of CN V or CN VII. Expansion of the skull base foramina and enlarged soft tissue density are suspicious findings on CT. MRI shows enlargement and an increased enhancement of CNs and foramina with various degrees of increased T2 signal, along with a loss of perineural fat on T1-weighted sequences (Figure 8) [35].

#### 4.1.6. Fibrous Dysplasia

**Figure 9:**
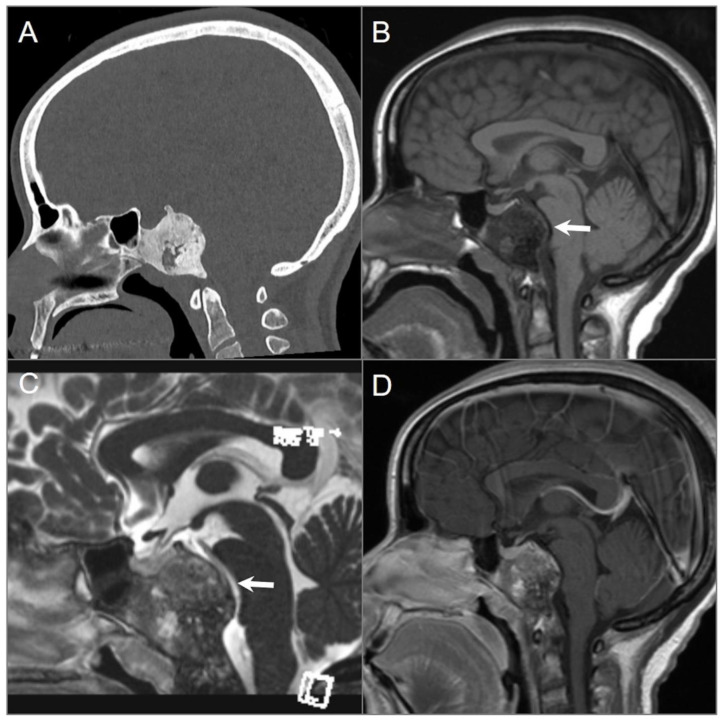
Fibrous dysplasia. (**A**) Sagittal CT image shows a well-defined expansile ground-glass density lesion in the clivus and posterior sphenoid sinuses. The lesion demonstrates peripheral sclerotic and central small lytic regions. (**B**,**C**) Sagittal T1W and postcontrast CISS images reveal T1 hypointense and variable T2 signal intensity in the lesion with mass effect on the brainstem and basilar artery (arrows). (**D**) Sagittal postcontrast T1-weighted image shows heterogeneous enhancement of the lesion.

Fibrous dysplasia is a developmental fibro-osseous disorder characterized by defective osteoblastic differentiation and maturation, causing the replacement of mature lamellar bone with fibrous tissue and immature woven bone. It is not considered a true neoplasm but is classified as a benign bony tumor in the WHO classification (fifth edition) [36]. Fibrous dysplasia can affect any bone, including those of the skull base, and may occur in a monostotic or polyostotic form. Lesions can exhibit varying and heterogeneous internal architecture, which can result in diverse imaging appearances on CT and particularly MRI. CT is the preferred imaging modality for diagnosing fibrous dysplasia and typically shows an expansile, homogenously sclerotic bone lesion with a ground-glass appearance. However, MRI can be difficult to interpret due to variations in bony trabecular composition, cellularity, collagen content, and cystic and hemorrhagic degeneration, which contribute to signal heterogeneity and may mimic a malignant or aggressive lesion (Figure 9) [37,38]. On FDG-PET CT, the lesions show variable and sometimes intense FDG uptake, which may be misinterpreted as metastases or malignant tumor [39].

#### 4.1.7. Aneurysmal Bone Cyst

Aneurysmal bone cysts (ABCs) are benign osteolytic vascular lesions characterized by bony expansion due to the partial occlusion and congestion of blood vessels. They can be locally destructive and most commonly affect the long bones of the limbs, vertebrae, and cranial bones. ABCs are rare in the skull base. They can develop de novo or secondary to other skull base lesions, such as giant cell tumors, and are typically diagnosed in adolescents [2]. The 2020 WHO Classification of Tumors of Bone introduced revised terminology, proposing the use of ‘ABC’ and ‘ABC-like changes’ instead of the previously used terms ‘primary ABC’ and ‘secondary ABC’, respectively. Over time, numerous theories have emerged to explain the pathophysiology of ABC. Lichtenstein’s 1950 hypothesis characterizes ABC as a reactive lesion triggered by local vascular disruption, resulting in increased intraosseous pressure, subsequent bone destruction, and expansion. Another theory suggests a traumatic origin, followed by an aberrant reparative process. Recent investigations have confirmed the presence of recurrent chromosomal translocations involving the USP6 gene, providing compelling evidence that supports the clonal neoplastic nature of both ABC and its solid variant [40,41,42,43].

CT usually shows an expansile lytic lesion with widened diploic spaces, cortical thinning, septations, and a well-defined thin margin that strongly enhances after contrast. On MRI, ABCs typically present as a well-defined, expansile, lobulated mass with internal septations and cysts that demonstrate low-to-medium signal intensity on T1WI and high signal intensity on T2WI [44,45]. The hallmark of ABCs is the presence of intracavitary fluid–fluid levels due to the layering of blood products in different stages of breakdown, best visualized on gradient echo T2WI. However, it is important to note that fluid–fluid levels are not specific to ABCs and may also be seen in other bone lesions such as osteosarcoma, chondroblastoma, fibrous dysplasia, and recurrent malignant fibrous histiocytoma [46,47]. Treatment options include sclerotherapy, embolization, curettage, and excision. Radiation therapy may be considered for lesions that are refractory to other treatment techniques, although it carries an increased risk of radiation-induced malignancies [2,48].

#### 4.1.8. Osteosarcoma

**Figure 10:**
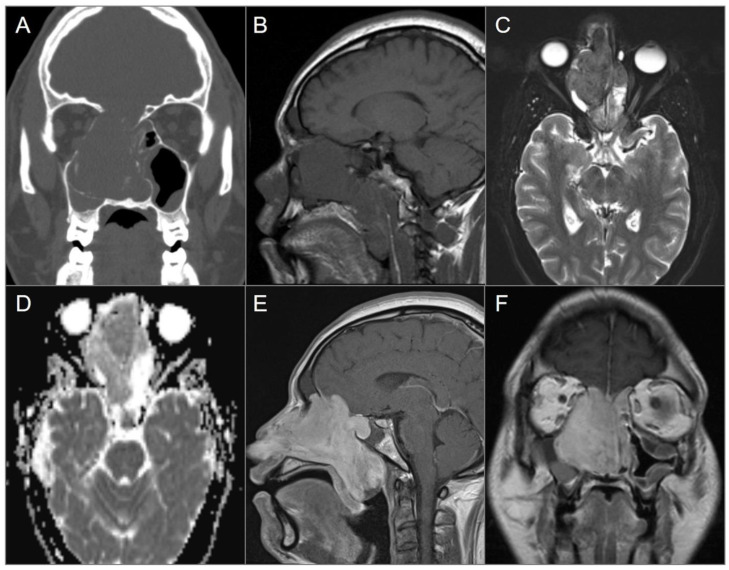
Nasal osteosarcoma invading skull base. (**A**) Coronal CT image shows a bulky expansile soft tissue density lesion in the nasal cavity, eroding the anterior skull base, nasal septum, and right lamina papyracea. (**B**) Sagittal T1W and (**C**) axial fat-saturated T2W MR images reveal slightly hypointense signal of the lesion on both sequences as well as mucus accumulation in the adjacent paranasal sinuses due to obstruction of the drainage pathways. (**D**) ADC map reveals mild diffusion restriction in the lesion anteriorly. (**E**) Sagittal and (**F**) coronal postcontrast T1W images demonstrate avid enhancement of the lesion with extension to the anterior cranial fossa, nasopharynx, and oropharynx.

Skull base osteosarcoma is a rare tumor that originates from osteoid-forming neoplastic cells. Unlike the classic primary osteosarcomas, which typically affect the long bones and are most commonly seen in adolescents males, skull base osteosarcomas tend to occur in the third decade of life and have no sex predilection. They also tend to be less aggressive [2,49,50]. Risk factors for secondary osteosarcoma include Paget’s disease, Li Fraumeni syndrome, fibrous dysplasia, and a history of cranial radiotherapy with a latent period of 4–50 years after irradiation. Lesions associated with prior radiation tend to be of higher grade [1]. CT often reveals a combination of lysis and sclerosis, with osteoid calcifications present in 75% of cases. Additionally, there may be a so-called Codman triangle or “sunburst” pattern of periosteal reaction. On MRI, the tumor has heterogeneous signal intensity on T1WI and T2WI, depending on mineral content. Osteosarcomas enhance avidly, following contrast administration, although usually to a lesser extent than chondrosarcomas (Figure 10) [1,2].

### 4.2. Anterior Skull Base Tumors

#### 4.2.1. Ossifying Fibroma

**Figure 11:**
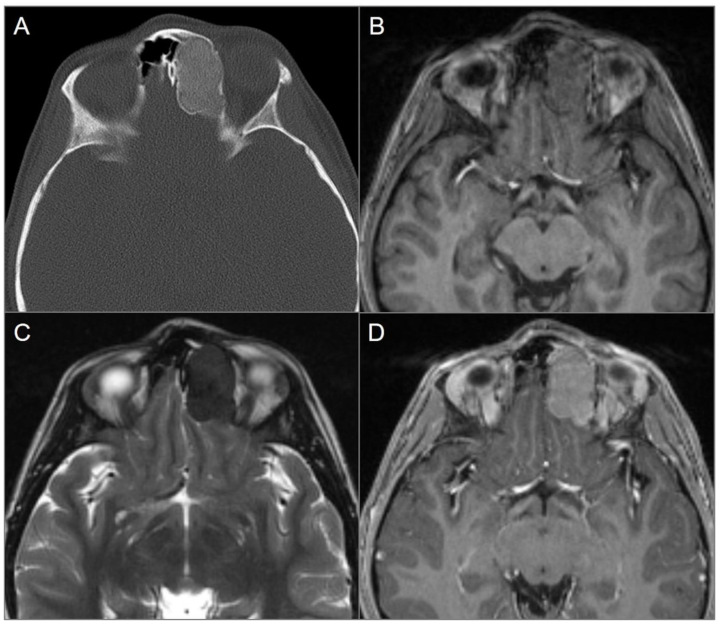
Ossifying fibroma. (**A**) Axial CT image shows an expansile and well-defined ground-glass density lesion with a thin sclerotic rim, centered in the left frontoethmoid sinuses. Note remodeling/scalloping of the left medial orbital wall and bulging into the left anterior cranial fossa. (**B**) T1W and (**C**) T2W axial images reveal isointense signal on T1 and markedly hypointense signal on T2. (**D**) Axial postcontrast T1W image demonstrates moderate homogenous enhancement of the lesion.

Ossifying fibromas are benign fibro-osseous lesions that primarily affect the mandible and maxilla, and may also extend to the nasal cavity and anterior skull base. They most commonly occur in females during the third and fourth decades of life. Although these lesions are often painless and detected incidentally, they can be locally aggressive, leading to nasal obstruction, proptosis, vision loss, or facial deformity [51]. The appearance of ossifying fibromas on CT and MRI depends on their internal composition, which varies with patient age. Lesions containing more fibrous tissue predominate in young children, while they gradually become more ossified as the patient ages. On CT, the typical finding is a well-delineated lesion with a fibrous center and a denser bony rim. On MRI, they usually demonstrate low-to-intermediate T1 signal and variable signal on T2WI related to its internal composition. The peripheral areas that are ossified typically show low signal, while the central areas that are non-ossified generally show a higher signal and contrast enhancement [2,39,51]. However, large and calcified masses may demonstrate an overall low signal on T2WI, which may be misdiagnosed as lymphoma or melanoma (Figure 11) [51]. Additionally, larger or more heterogeneous lesions may be difficult to distinguish from fibrous dysplasia [2,39].

#### 4.2.2. Osteoma

**Figure 12:**
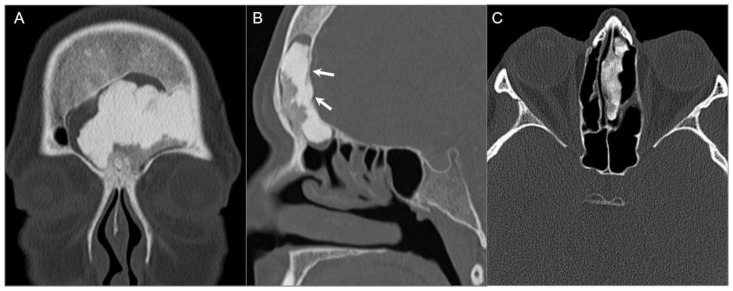
Osteomas in two different patients. (**A**) Coronal and (**B**) sagittal CT images show a large frontal sinus osteoma with mixed dense and hypodense areas consistent with cortical and cancellous components, respectively. Note mild intracranial extension (arrows). (**C**) Axial CT image in a different patient demonstrates a well-defined, heterogeneously hyperdense lesion centered in the left ethmoid sinus and attached to the nasal septum.

Osteomas are benign primary bone tumors characterized by the focal growth of mature bone from osseous structures of the skull base and walls of the paranasal sinuses. They are most frequently encountered in the frontal sinus. Osteomas occurring in the osseous medullary cavity are also called bone islands or enostoses. They are commonly discovered incidentally and are usually asymptomatic due to their small size and slow growth. However, lesions that cause the blockage of paranasal sinus drainage pathways can present with headaches and sinusitis. Rarely, lesions can grow enough to erode into the intracranial space. There are two distinct types of osteomas: cortical osteoma, which contains predominantly cortical bone, and cancellous osteoma, which contains predominantly cancellous bone. Cortical osteomas are hyperdense on CT and hypointense on all MRI sequences due to compact bone. In contrast, cancellous osteomas show central bone marrow signal intensity on MRI and low signal in the periphery due to compact bone (Figure 12). The majority of osteomas are asymptomatic and require no treatment, but symptomatic osteomas are typically surgically excised. Patients with multiple peripheral osteomas should be investigated for Gardner’s syndrome due to its premalignant potential. Gardner’s syndrome consists of multiple osteomas, supernumerary teeth, odontomas, dentigerous cysts, familial adenomatoid gastrointestinal polyps, and epidermoids [2,52].

#### 4.2.3. Olfactory Neuroblastoma

Olfactory neuroblastoma is a primary neuroectodermal tumor that arises from the basal layer of olfactory epithelium in the upper nasal fossa. It accounts for 3–5% of all intranasal neoplasms and has bimodal incidence peaks in childhood and in individuals over 50 years old, without gender predilection. The typical clinical presentation is nasal obstruction (70%) and mild epistaxis (46%) [5,53].

Olfactory neuroblastoma typically presents as a soft tissue mass in the superior olfactory recess at the level of cribriform plate, with downward extension involving the anterior and middle ethmoid air cells and upward extension into the anterior cranial fossa. In some cases, the tumor may remain completely extracranial and extend into the orbit. In 1976, Kadish et al. introduced a clinical staging system that continues to be an important predictor of prognosis. Generally, tumors classified as stage A and B demonstrate excellent survival rates, while tumors that invade the cribriform plate or extend to the orbits have a poor prognosis. Later, Morita proposed a revised staging system that accounts for local disease extending beyond the paranasal sinuses (stage C) and cervical or distant metastases (stage D). While other TNM-based staging systems have been developed, they have not gained widespread acceptance due to a lack of evidence supporting their ability to provide better prognostic differentiation [54,55,56,57,58]. Treatment includes surgery and chemotherapy and/or radiation therapy, and lesions are associated with a high rate of local recurrence [53].

**Figure 13:**
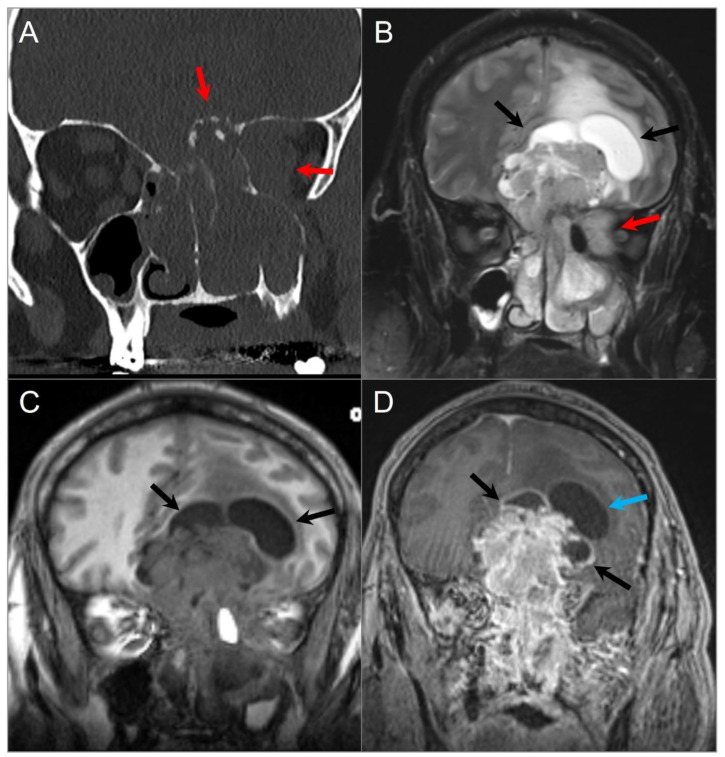
Olfactory neuroblastoma. (**A**) Coronal CT image shows a soft tissue density lesion centered in the left greater-than-right superior nasal cavity with left intraorbital and anterior cranial fossa extension through erosion of the cribriform plate and lamina papyracea (red arrows). (**B**) T2W and (**C**) noncontrast T1W coronal images reveal a dumbbell-shaped mass with heterogeneous signal intensity extending into the superior nasal cavity, paranasal sinuses, anterior cranial fossa, and left orbit (red arrows). The ‘waist’ of the dumbbell is centered at the cribriform plate. The mass causes marked mass effect on the adjacent frontal lobes with vasogenic edema. Peritumoral cysts are also noted at the tumor–brain interface (black arrows). (**D**) Coronal postcontrast T1W image shows intense heterogeneous and irregular enhancement of the lesion with smooth wall enhancement of the several peritumoral cysts, while one shows no appreciable enhancement (blue arrow).

Olfactory neuroblastoma appears iso- to slightly hyperdense on noncontrast CT with erosion of the cribriform plate when there is intracranial extension. Focal intratumoral calcifications may occasionally be seen. On MRI, the tumor appears as an ill-defined, heterogenous mass with intermediate T1 and T2 signal intensity as well as variable enhancement that is usually moderate to intense. Peritumoral cysts at the tumor–brain interface may be present when the tumor extends intracranially, which is highly suggestive of olfactory neuroblastoma (Figure 13) [2,59,60].

### 4.3. Central Skull Base Tumors

#### 4.3.1. Intraosseous/Ectopic Pituitary Adenoma

**Figure 14:**
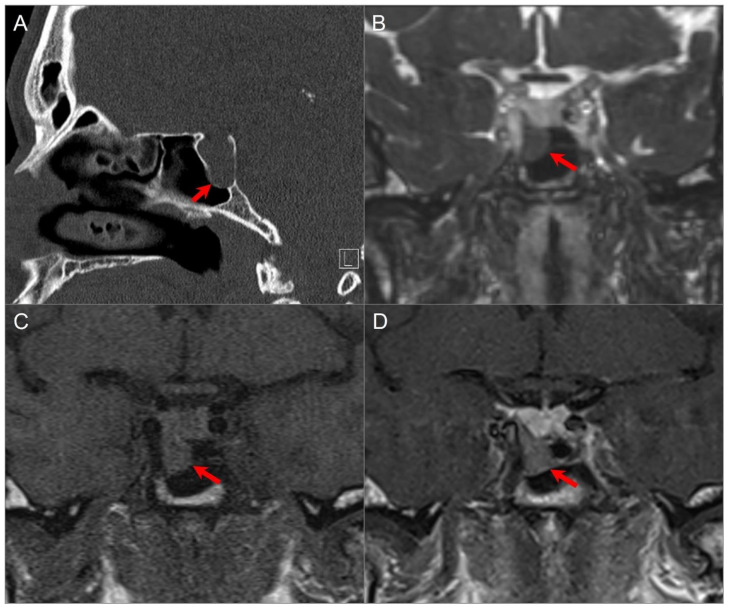
Exophytic/ectopic-intrasphenoidal pituitary adenoma. (**A**) Sagittal CT image shows erosion of the sellar floor with a soft tissue lesion extending into the clivus and posterior margin of the sphenoid sinus (red arrow). (**B**) Coronal postcontrast CISS and (**C**) noncontrast T1W images reveal a hypointense lesion abutting the inferior margin of the right pituitary lobe and medial wall of the petrocavernous ICA. (**D**) Coronal postcontrast T1-weighted image demonstrates normal enhancement of the pituitary gland and hypoenhancement of the exophytic adenoma (red arrow).

**Figure 15:**
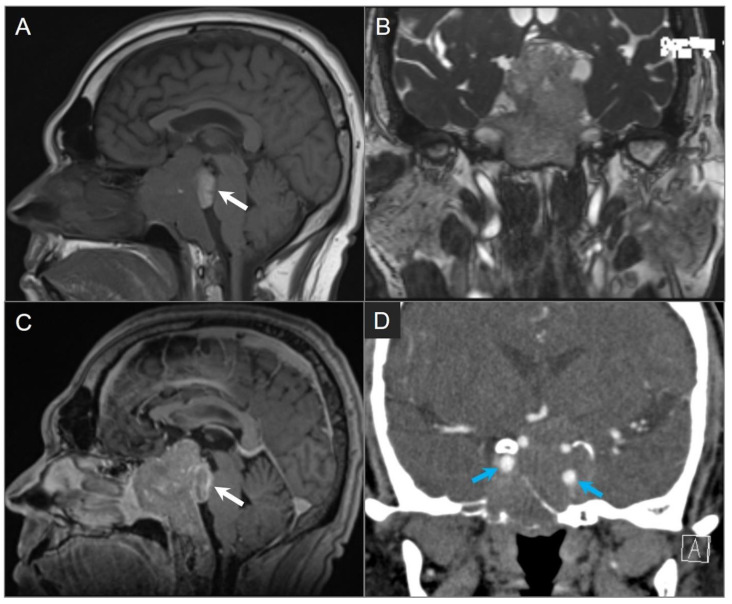
Giant invasive macroadenoma. (**A**) Sagittal noncontrast T1W, (**B**) coronal postcontrast CISS, and (**C**) sagittal postcontrast T1W MR images show tumor invading the clivus, cavernous sinuses, and posterior sinonasal cavity. Probably hemorrhagic T1 hyperintense component of the tumor extends into the prepontine cistern and compresses the pons (white arrow) with mass effect on the optic chiasm and third ventricle. (**D**) Coronal CTA shows encasement of the left greater-than-right cavernous internal carotid artery without significant vascular narrowing (blue arrows).

The adenohypophysis develops from the embryonic Rathke pouch, which originates at the inferior margin of the sphenoid and migrates through it to the sella turcica. Normally, the craniopharyngeal canal regresses, but in rare cases, it persists along with some adenohypophyseal remnants. Ectopic pituitary adenomas may occur within the sphenoid sinus, clivus, and nasopharynx along the expected course of the craniopharyngeal canal (Figure 14). The classical signal intensities of a pituitary macroadenoma also apply to this unusual form: hypointense on T1WI, hyperintense on T2WI, some degree of contrast enhancement, and surrounding smooth bony remodeling without clear connection between the lesion and the sella turcica [61]. Although the majority of pituitary adenomas have a benign histology, they can be biologically aggressive and infiltrate the skull base, clivus, and sphenoid sinus (Figure 15). In rare cases, tumors may extend to the nasopharynx [62]. Intraosseous pituitary adenomas, on the other hand, tend to be limited by the sphenoid cortices and the petro-occipital synchondroses. This feature can be useful in distinguishing them from plasmacytomas, which appear as smoothly delineated mass lesions capable of crossing the synchondroses [2].

#### 4.3.2. Craniopharyngioma

Craniopharyngiomas are tumors of nonglial epithelial origin that arise from remnants of Rathke’s pouch or the rests of buccal mucosa at any point along the trajectory of the craniopharyngeal duct [6,63]. The craniopharyngeal canal extends from the floor of the sella to the vomer and may give rise to ectopic craniopharyngiomas. Infrasellar craniopharyngiomas are rare and most commonly arise in the sphenoid sinus, either alone or in combination with other sites such as the nasopharynx, sella turcica, suprasellar region, ethmoid sinuses, or maxillary sinus. There is a bimodal age distribution with peaks in childhood and the fourth to fifth decades of life [64,65].

**Figure 16:**
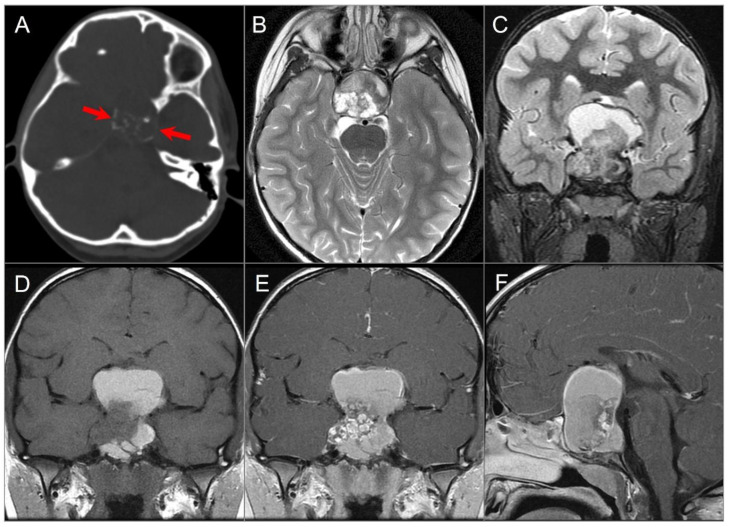
Adamantinomatous craniopharyngioma in a child. (**A**) CT image shows a partially calcified sellar and suprasellar mass (red arrows). (**B**) Axial and (**C**) coronal T2W, (**D**) coronal noncontrast T1W, and (**E**) coronal and (**F**) sagittal postcontrast T1W images show a complex mass with mixed cystic and solid components. Note: hyperintense T1 and T2 signal in the cystic components consistent with proteinaceous, hemorrhagic and/or cholesterol contents. The mass demonstrates intrasphenoidal and intraclival extension with mild prepontine bulge and suprasellar extension with mass effect on the optic chiasm and third ventricle. Heterogenous enhancement of the solid tumor components is noted.

Histologically, craniopharyngiomas are almost always benign WHO grade 1 tumors with a high survival rate. However, they can be locally aggressive and associated with significant morbidity [66]. Those occurring in childhood are more commonly of the adamantinomatous type and present as heterogeneous cystic and solid masses. Approximately 90% of these have calcifications that can be readily identified on CT [63,66]. The solid portions show contrast enhancement, and on MRI, the cystic components may present with variable signal intensities depending on their contents of protein, cholesterol, or hemorrhage (Figure 16) [63]. Tumors in adulthood are more commonly of the papillary type and are solid, less commonly calcified, and devoid of cysts [63,66].

Complete surgical excision is the treatment of choice and is easier to achieve for infrasellar craniopharyngiomas as they do not intimately involve sellar and suprasellar structures such as the optic apparatus or hypothalamus.

#### 4.3.3. Langerhans Cell Histiocytosis

Langerhans cell histiocytosis (LCH) is a rare multisystem disease with a wide clinical spectrum and varying degrees of organ involvement. The disease results from the uncontrolled monoclonal proliferation of Langerhans cells outside the dermis and is considered an inflammatory myeloid neoplasia, although its biological behavior is variable [67,68,69].

LCH encompasses three entities historically known as eosinophilic granuloma, Hand–Schuller–Christian disease, and Letterer–Siwe disease, although their definitions can be relatively confusing. LCH is more prevalent in the pediatric population, with a peak incidence between the ages of 1–3 years and a slight male predominance [67]. Although LCH can affect any region of the skull base and orbits, the temporal bone is the most commonly affected site [2].

**Figure 17:**
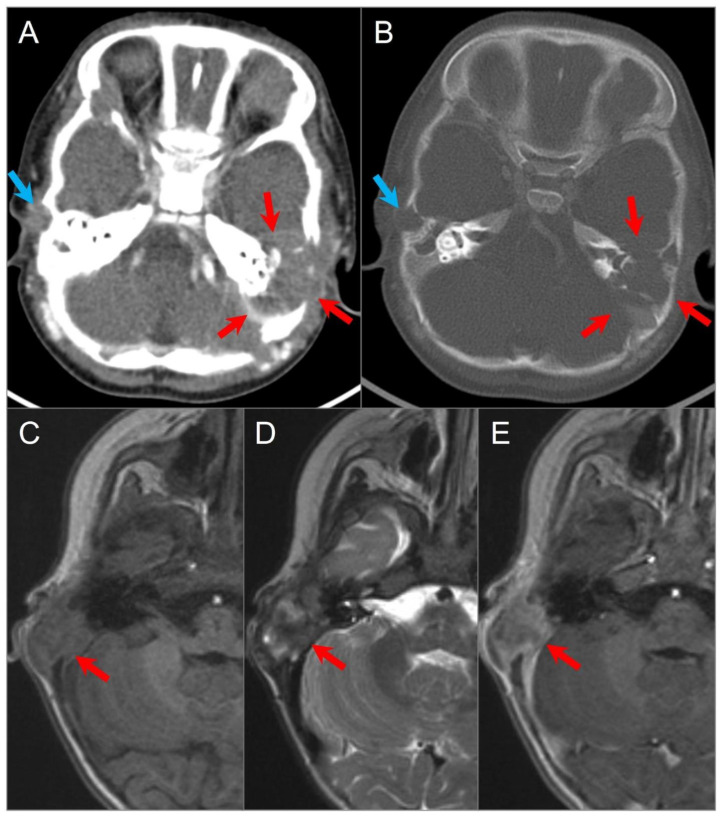
Langerhans cell histiocytosis in a 9-month-old boy. Axial postcontrast CT in soft tissue (**A**) and bone window (**B**) settings shows a lytic ‘punched out’ bone lesion involving the left petromastoid temporal bone with middle and posterior cranial fossa extensions (red arrows). The lesion also erodes the left semicircular canals and involves the left middle ear cavity and facial nerve. Another small destructive lesion with similar CT characteristics is seen in the right temporal bone (blue arrows). In a different 3-year-old boy, (**C**) noncontrast T1W, (**D**) T2W, and (**E**) postcontrast T1W axial images demonstrate a relatively well-delineated lesion in the right temporal bone (red arrows). The lesion demonstrates a slightly heterogeneous isointense signal on T1 and mixed hypointense and hyperintense signals on T2WI with heterogeneous enhancement after gadolinium administration.

CT typically reveals destructive, lytic “punched out” bone lesions, while MRI is better able to demonstrate a soft tissue component and shows a sharply delineated mass with various T1 and T2 signal intensities. Lesions can appear profoundly hypointense on T2WI, possibly due to high cellularity. Following contrast administration, CT and MRI both show a homogeneous enhancement of the LCH lesion and associated soft tissue components (Figure 17) [68,69]. Children with localized or multifocal LCH who do not have organ dysfunction generally have an excellent prognosis [2].

#### 4.3.4. Cholesterol Granuloma

Cholesterol granulomas are cystic tumors that contain granulation tissue, blood products, cholesterol crystals, and multinucleated giant cells covered by a fibrous pseudocapsule. These lesions can be found in any aerated portion of the temporal bone, including petrous apex, middle ear cavity, and mastoid, with the mastoid air cells being the most common location. The pathogenesis is controversial, but the most accepted hypothesis is the obstruction-vacuum theory characterized by eustachian tube dysfunction, resulting in a chronic ventilation outlet obstruction that leads to recurrent mucosal inflammation and hemorrhage. Hemoglobin extravasations and cholesterol crystals degraded by hemoglobin cause irritation and trigger macrophage activity, resulting in giant cells and granuloma formation.

**Figure 18:**
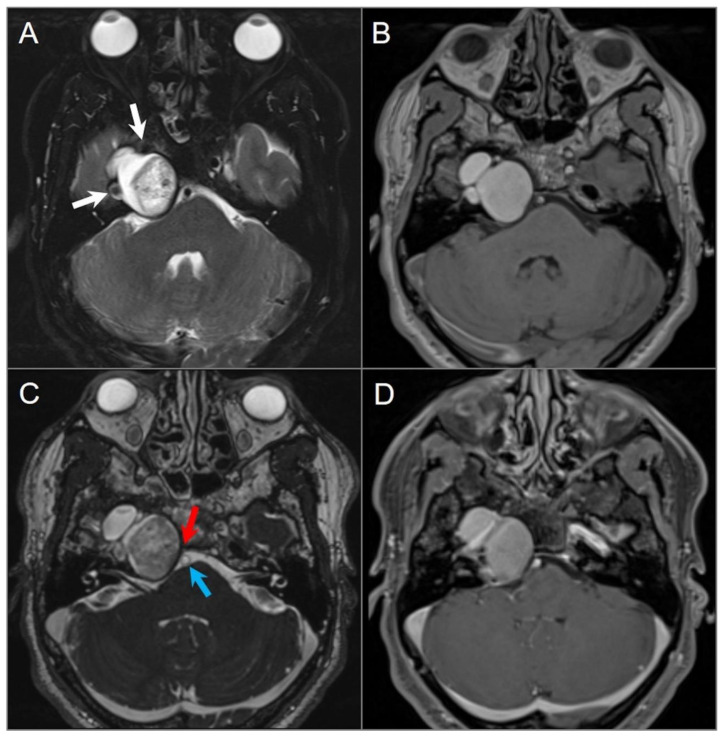
Cholesterol granuloma. (**A**) Axial fat-suppressed T2W and (**B**) noncontrast T1W images show a lobulated, expansile lesion in the right petrous apex with intrinsic high signal intensity on T1 and mixed signal intensity with smooth hypointense margins on T2. The lesion is in close proximity with the petrous ICA (white arrow). (**C**) Axial CISS image shows extension of the lesion into the right cerebellopontine angle with mild mass effect on the pons and abutment of the basilar artery (blue arrow). The lesion effaces right Dorello’s canal, where there is mass effect with effacement of the cisternal segment of the right sixth cranial nerve (red arrow). (**D**) Axial fat-saturated postcontrast T1-weighted image demonstrates no significant enhancement of the lesion, although this is difficult to evaluate due to intrinsic T1 hyperintensity.

CT typically demonstrates an expansile, well-marginated, and rounded lesion with bone remodeling and cortical thinning [2]. Expanding lesions can cause bony erosion, most commonly in the petrous apex and rarely in the middle ear. On MRI, the lesion demonstrates intrinsic T1 and T2 signal hyperintensities due to cholesterol crystals, methemoglobin, and proteinaceous debris without fat suppression or diffusion restriction. At times, hemosiderin deposition at the periphery can cause a T1 and T2 hypointense rim, and there may be internal T2 hypointense areas due to prior hemorrhage. The lesion does not enhance centrally, but reactive peripheral enhancement can be present, although difficult to depict due to the intrinsic high T1 signal of lesion (Figure 18) [2,70]. Cholesterol granulomas are slow-growing lesions and can be stable and asymptomatic for long periods. However, surgical excision, including the cyst wall, is required in symptomatic and rapidly progressive cases [71].

### 4.4. Posterior Skull Base Tumors

#### 4.4.1. Chordoma

Chordomas are rare tumors that originate from embryonic remnants of the primitive notochord, which represents the earliest fetal axial skeleton extending from the Rathke’s pouch to the tip of the coccyx. They can occur anywhere from the clivus to the coccyx and are usually seen between the age of 30–50 years. Following sacrococcygeal involvement, the clival region is the second most common location, accounting for 30–35% of cases [72,73]. Chordomas are locally aggressive and cause bone destruction, but they rarely metastasize. The typical appearance is a midline mass lesion projecting posteriorly and indenting the pons in patients with intracranial extension, a finding termed the “thumb sign”. Chordomas demonstrate a slow-growing pattern and exert mass effect on adjacent structures such as the brainstem, CNs, nasopharynx, and spinal cord [73].

**Figure 19:**
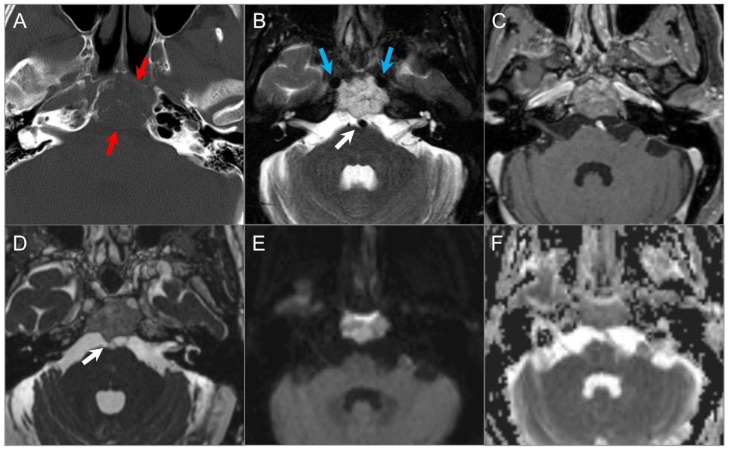
Chordoma. (**A**) Axial CT image shows a well-defined lytic lesion in the clivus (red arrows) with associated erosion and minimal intratumoral calcifications. (**B**) Axial fat-suppressed T2W image reveals a marked T2 hyperintensity of the mass with linear hypointense septations, resulting in a ‘soap bubble’ appearance. Note: abutment of the bilateral ICAs (blue arrows). (**C**) Axial postcontrast T1W image demonstrates heterogeneous enhancement of the lesion. (**D**) Axial CISS image demonstrates bulging into the prepontine cistern and abutment to basilar artery (white arrow) without definite dural disruption. (**E**) DWI demonstrates increased signal and (**F**) ADC map shows similar diffusion characteristics with brain parenchyma in the lesion.

CT shows a centrally located, well-circumscribed, and destructive lytic retroclival mass with osseous erosion and, sometimes, marginal sclerosis. Lesions frequently contain coarse calcifications that reflect a sequestra of normal bone, rather than dystrophic calcifications. On MRI, lesions appear lobulated with well-defined margins, often centered at the level of the spheno-occipital synchondrosis. On T1-weighted images, the tumor is predominantly hypointense with varying degrees of heterogeneous hyperintensity due to intratumoral calcifications, hemorrhage, and/or mucoid components [2,72,73]. On T2WI, it shows a very high signal intensity, which can be higher than CSF and with a “soap bubble” appearance. Enhancement is variable and often appears inhomogeneous with a honeycomb pattern, which is caused by epithelioid cells surrounding lakes of mucinous material or necrosis (Figure 19) [74,75].

#### 4.4.2. Chondrosarcoma

Chondrosarcomas are rare malignant tumors that arise from endochondral cartilage remnants along the skull base synchondroses. They constitute only 2% of all chondrosarcomas [2] and can be associated with prior trauma and several syndromes, including Ollier disease, Maffucci syndrome, and Paget disease, although most cases are sporadic. More than 80% of skull base chondrosarcomas occur off midline, most commonly involving the petro-occipital synchondrosis, followed by the sphenoethmoidal junction and sella turcica. They can rarely occur at distant sites due to the metaplasia of the dura mater, arachnoid mater, and choroid plexus. Primary presenting symptoms are headache and neurologic deficits caused by the compression of the CNs and brainstem. Skull base chondrosarcomas are generally low-grade malignancies and prognosis is good following excision with or without radiation therapy [2,6].

**Figure 20:**
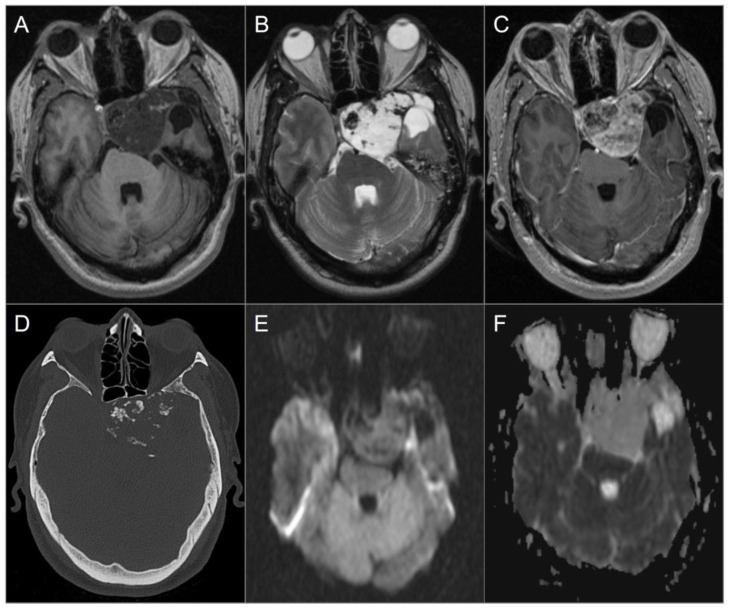
Chondrosarcoma. (**A**) Axial noncontrast T1W and (**B**) T2W images show a well-circumscribed T1 hypointense, markedly T2 hyperintense lesion centered in the left petroclival fissure involving the clivus, cavernous sinus, posterior aspect of the sphenoid sinus and left middle cranial fossa. The lesion extends into the prepontine cistern and compresses the brain stem, left temporal lobe, and basilar artery. (**C**) Axial postcontrast T1W image shows avid heterogeneous enhancement of the mass. (**D**) Axial CT image shows permeative destructive margins and chondroid calcifications in the lesion. (**E**) DWI and (**F**) ADC map images reveal facilitated diffusion in the lesion.

Chordomas and chondrosarcomas have a similar appearance on CT and MRI and can be difficult to distinguish. In general, chondrosarcomas arise at paramedian fissures along the skull base, whereas chordomas occur midline along the notochordal remnant. On CT, chondrosarcomas appear as well-defined lytic lesions with permeative destructive margins and chondroid matrix calcifications in about half of the cases. In contrast, calcifications in some chordomas represent fragments of destroyed bone, although the distinction may be difficult. On MRI, both tumors have low-to-intermediate signal intensity on T1WI and high signal intensity on T2WI. However, T2 hyperintensity tends to be more prominent in chondrosarcomas due to the presence of a chondroid matrix. Both tumors show variable moderate-to-intense heterogenous contrast enhancement [6,76,77,78]. DWI may be useful in distinguishing between the two, with chondrosarcoma showing high ADC values, and classic and poorly differentiated chordomas showing low ADC values due to high cellularity (Figure 20) [79].

#### 4.4.3. Plasmacytoma

Solitary skull base plasmacytoma is a rare tumor characterized by the localized proliferation of neoplastic monoclonal plasma cells. Although tumors are histologically similar to the bone lesions found in multiple myeloma, the latter is a systemic disease. Plasmacytoma is divided into two distinct forms: extramedullary plasmacytoma and intramedullary solitary plasmacytoma of bone. The intramedullary form is more common and may occasionally be multiple [80].

**Figure 21:**
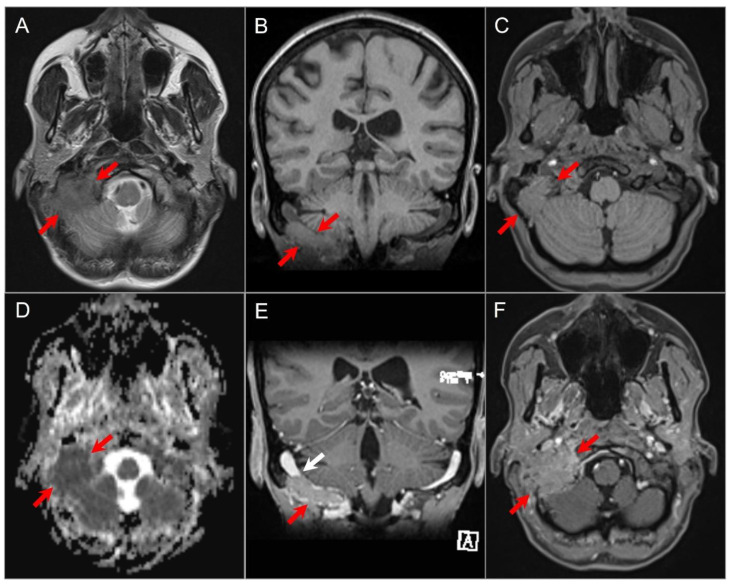
Plasmacytoma. (**A**) Axial T2W image shows a soft tissue mass lesion involving the right occipitomastoid region with predominantly iso- to slightly hypointense signal (white arrows). (**B**) Coronal and (**C**) axial noncontrast T1W images show the mass to be slightly hyperintense relative to the cerebral cortex (red arrows). (**D**) ADC map reveals restricted diffusion in the lesion consistent with high cellularity. (**E**) Coronal and (**F**) axial postcontrast T1W images show diffuse homogenous enhancement of the mass and invasion of the right sigmoid sinus (white arrow). By imaging, this mass is indistinguishable from a metastasis.

Skull base plasmacytomas most commonly arise from the clivus and sphenoclival region, followed by the nasopharynx, petrous apex, and orbital roof [81]. Accurate diagnosis is crucial as the treatment and prognosis of plasmacytomas differ from other skull base lesions. However, the imaging appearance is nonspecific and generally does not allow for a preoperative diagnosis. The most common presentation on CT is a slightly hyperdense lytic lesion with non-sclerotic margins in the diploic space. On MRI, the lesion is isointense to gray matter on T1WI and T2WI and shows avid and homogeneous contrast enhancement. Some tumors may demonstrate slight intrinsic T1 hyperintensity, which suggests the diagnosis and is likely related to densely packed cells and low water content (Figure 21) [82]. Relative T2 isointensity is helpful in differentiating it from chondrosarcomas or chordomas, which are markedly hyperintense on T2WI [82,83].

#### 4.4.4. Endolymphatic Sac Tumor

**Figure 22:**
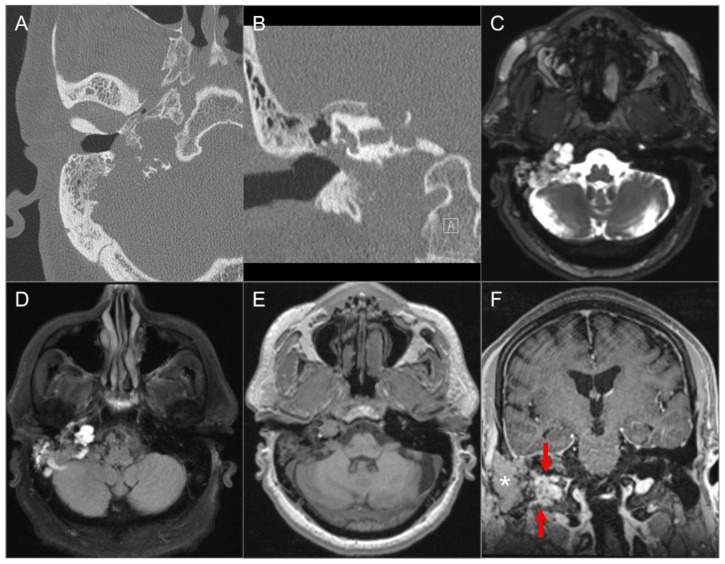
Endolymphatic sac tumor. (**A**) Axial and (**B**) coronal CT images show a lytic lesion with a moth-eaten, permeative appearance in the right retro-labyrinthine petrous temporal bone extending to the middle ear cavity, mastoid region, and jugular fossa. (**C**) Axial fat-suppressed T2W, (**D**) axial FLAIR, and (**E**) axial noncontrast T1W images demonstrate a complex solid and cystic lesion with mixed signal intensity, likely reflecting hemorrhagic and proteinaceous contents. (**F**) Postoperative coronal postcontrast T1-weighted image shows heterogeneous enhancement in the residual solid component of the lesion (red arrows). The more lateral tissue corresponds to a fat graft (asterisk).

Endolymphatic sac tumor is a rare, highly vascular, and locally aggressive papillary cystadenoma originating from the epithelium of the endolymphatic sac and duct along the posterior aspect of the petrous temporal bone. It is typically encountered in young patients, with an average age of 22 years at presentation, and is associated with symptoms such as hearing loss, tinnitus, dizziness and facial nerve palsy. Tumors can be found in up to 15% of individuals affected with VHL disease, with bilateral occurrence in approximately 30% of patients [84,85,86]. Sensorineural hearing loss is often the presenting symptom, which can be caused by several factors such as intratumoral hemorrhage extending to the labyrinth, endolymphatic hydrops due to reduced endolymph resorption, excessive inflammatory response to hemorrhage, and otic capsule invasion [2]. Therefore, early detection is critical since early surgical intervention can prevent further hearing loss. Proximity to the vestibular aqueduct is a key diagnostic feature. Although the tumor does not metastasize, it is locally aggressive and can invade the mastoid, semicircular canals, CPA, and CNs, findings commonly present at diagnosis. On CT, typical imaging findings include the erosion of the petrous bone in an infiltrative or “moth-eaten” pattern, dilatation of the vestibular aqueduct, intratumoral calcifications, and usually intense enhancement. On MRI, intrinsic foci of T1 hyperintensity due to hemorrhagic and proteinaceous content, heterogeneous T2 signal, and enhancement in solid components are typical (Figure 22) [87].

#### 4.4.5. Paraganglioma

Glomus jugulare and tympanicum, also known as paragangliomas, are tumors that develop from glomus bodies near Jacobson’s nerve (tympanic branch of CN IX) and Arnold’s nerve (auricular branch of CN X). They typically affect adults aged between 40 and 60 years and are more common in women [2,88]. Patients can present with an isolated primary jugular fossa paraganglioma, known as glomus jugulare, or may have a tumor that extends to the middle ear along Jacobson’s nerve, referred to as glomus jugulotympanicum. Glomus tympanicum classically arises from the cochlear promontory, and it can be seen as a small red mass in the anterior inferior quadrant of the tympanic membrane during otoscopy. Up to 10% of patients may have multiple paragangliomas, particularly when occurring in association with succinate dehydrogenase gene mutations [2,89].

The clinical presentation can vary depending on the degree of involvement of the jugular fossa and middle ear and can include symptoms such as pulsatile tinnitus, hearing loss, and CN palsies. Assessing the extent of bony erosion, an involvement of the middle ear cavity, and intracranial extension are critical for surgical planning. CT is the modality of choice to demonstrate the erosion of adjacent bony structures, including the jugular and caroticojugular spines. CT is also helpful to determine the degree of extension into the middle ear cavity and infratemporal fossa as well as the integrity of the ossicles and bony labyrinth [88,90,91].

**Figure 23:**
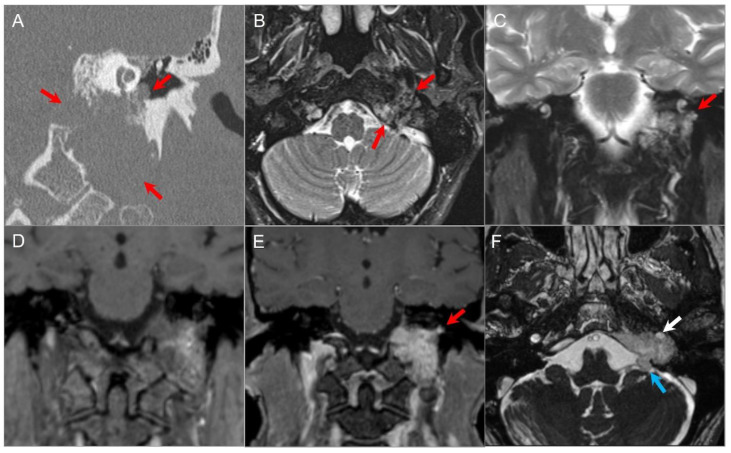
Glomus jugulotympanicum. (**A**) Coronal CT image demonstrates a soft tissue density lesion in the left jugular fossa with irregular moth-eaten osseous erosion. The mass extends into the middle ear cavity at the cochlear promontory (red arrows), confirming a combined glomus jugulotympanicum. (**B**) Axial and (**C**) coronal fat-suppressed T2W images demonstrate heterogeneous hyperintense signal in the lesion (red arrows) with tiny signal voids consistent with hypervascularity. (**D**) Coronal noncontrast T1W image demonstrates a ‘salt and pepper’ appearance with small foci of hyper- and hypointensity. (**E**) Coronal postcontrast T1W image shows avid enhancement of the lesion (red arrow). (**F**) Axial postcontrast CISS image reveals partial encasement of the left ICA (white arrow) and polypoid extension of the lesion into the left CPA cistern (blue arrow) due to dural disruption.

These tumors are highly vascular and can be assessed by DSA and MRA, which show an intense tumor blush, feeding vessels primarily from the ascending pharyngeal artery, and early venous drainage due to intratumoral shunts. On MRI, a “salt and pepper” appearance can be seen on T1WI and T2WI, where the “salt” represents blood products from hemorrhage or slow flow and the “pepper” represents flow voids due to high vascularity. Intense enhancement is typical on both CT and MRI (Figure 23) [88,91]. Surgery is the primary treatment, but large inoperable tumors or those in poor surgical candidates can be treated with radiotherapy as these lesions are radiosensitive [2,89].

## 5. Skull Base Tumor Mimics: Developmental Lesions, Anatomic Variations and Pseudotumors

### 5.1. Arrested Pneumatization

**Figure 24:**
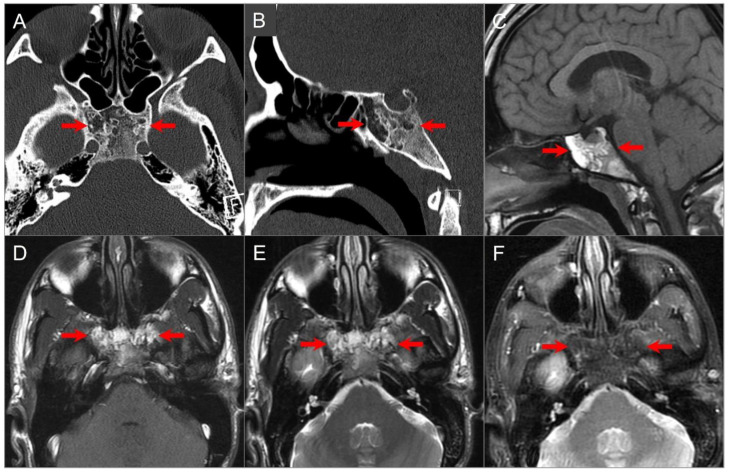
Arrested pneumatization. (**A**) Axial and (**B**) sagittal CT images show partial pneumatization of the sphenoid sinuses anteriorly with a heterogeneous appearance of the sphenoid body. There are multiple hypodense foci with peripheral sclerosis, a non-expansile and non-destructive appearance, and similar density to subcutaneous fat (red arrows). (**C**) Sagittal and (**D**) axial noncontrast T1W and (**E**) axial T2W images reveal heterogeneous hyperintense signals in these regions. (**F**) Fat-suppressed T2W image shows signal suppression consistent with fatty bone marrow.

Arrested pneumatization presents as a focal variation of the marrow pattern within the central skull base and is a benign finding does not require intervention. It is important to recognize this entity as it is common and can be mistaken for pathology within the sphenoid sinus. The development of the sphenoid sinus begins at the age of 2 years and typically reaches adult size by the age of 14 years [2,92,93]. Disruption of this process can lead to areas of reduced pneumatization within the skull base. On CT, this appears as a well-defined non-expansile area with a decreased attenuation in the body of the sphenoid, commonly with fat attenuation and relatively sclerotic borders. MRI may show central areas of T1 hyperintensity depending on the amount of fatty marrow. Lesions typically lose signal on fat-saturated sequences; however, they can contain microcystic components that appear hyperintense on T2WI (Figure 24). There is no significant enhancement after the administration of intravenous contrast [2,92].

### 5.2. Asymmetric Petrous Apex Pneumatization

Asymmetric pneumatization of the petrous apex is a frequent normal anatomic variant that can be mistaken for pathology. The petrous apex normally contains bone marrow in 60% of patients, is pneumatized in 33%, and is sclerotic in 7%. However, in 5–10% of patients, pneumatization is asymmetric.

Asymmetric fatty infiltration of the petrous apex can result in conspicuous asymmetric high signal on T1WI and T2WI, which can be misdiagnosed as a contrast-enhancing lesion or a cholesterol granuloma. Correlation with intrinsic T1 hyperintensity on pre-contrast T1WI, fat suppression, and lack of expansion, enhancement, or bone destruction can aid in differentiation [71,94].

### 5.3. Arachnoid Granulations

Arachnoid granulations are small protrusions of arachnoid tissue that extend through the dura mater into a dural venous sinus, where they drain CSF from the subarachnoid space into the bloodstream. They consist of a core of loose connective tissues that form a trabeculated network with wide interstices and endothelium-lined channels [95,96]. Aberrant arachnoid granulations are those that penetrate the dura but fail to migrate normally into the venous sinus. They are often found in the greater wing of the sphenoid bone and occasionally in the posterior temporal bone, and may be seen in idiopathic intracranial hypertension. Generally, they are an incidental finding, but in some cases, they can enlarge and lead to CSF leakage or cephaloceles. Rarely, infection from the mastoid can spread intracranially through these structures [2].

Aberrant posterior temporal arachnoid granulations may sometimes mimic an enlarged endolymphatic sac or endolymphatic sac tumor. However, their sharply demarcated lobulated contours and signal properties on MRI, which are similar to CSF on T1WI and T2WI, are essential for accurate diagnosis. In contrast, endolymphatic sac tumors show heterogeneous T1 and T2 signal with focal T1 hyperintensities due to hemorrhagic and proteinaceous components, and they also enhance heterogeneously on postcontrast images. Occasionally, adjacent brain tissue may herniate into giant arachnoid granulations, resulting in gliosis with areas of T2 and FLAIR hyperintensities [2].

### 5.4. Cephaloceles

**Figure 25:**
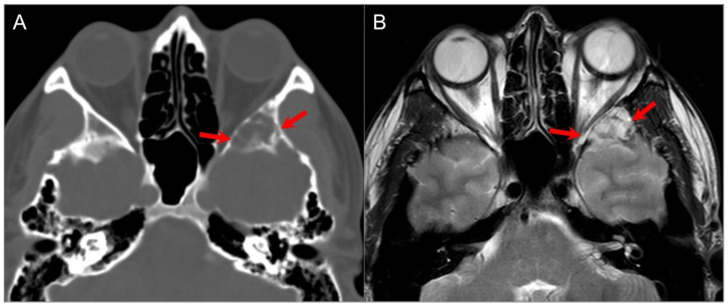
Sphenoid wing cephalocele. (**A**) Axial CT image reveals a lobulated, well-defined lucent lesion in the left greater sphenoid wing with bony scalloping and thin internal septations (red arrows). (**B**) T2W axial MR image shows CSF isointense signal within the lesion with linear hypointense septations.

**Figure 26:**
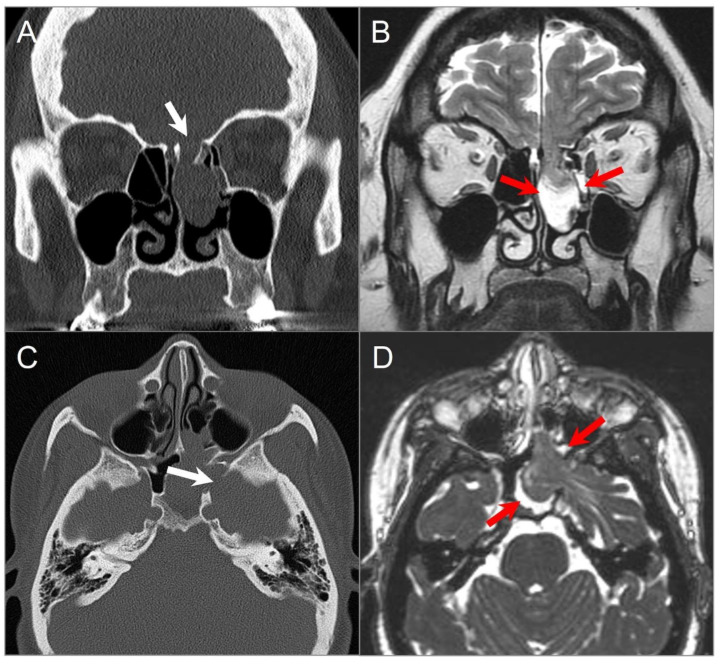
Ethmoidal and sphenoidal menigoencephaloceles. (**A**) Coronal CT image reveals a bony defect in the left cribriform plate (white arrow), with a well-defined polypoid lesion protruding into the left-sided ethmoid sinus and nasal cavity. (**B**) Coronal T2W image reveals that the lesion correlates with herniated left frontal lobe parenchyma, CSF, and meninges through the bony defect (red arrows). (**C**) Axial CT image in a different patient demonstrates a bony defect in the left lateral sphenoid sinus wall (white arrow), with opacification of the left sphenoid sinus and left posterior ethmoid air cells. (**D**) Axial CISS image shows herniation of the anteromedial left temporal lobe, meninges, and CSF through the bony defect (red arrows).

Cephaloceles can be classified into meningoceles, where there is a herniation of meninges and CSF through a skull defect, and encephaloceles, which contain additional herniation of brain tissue. These are more commonly seen in the anterior skull base where they may be congenital or due to preceding trauma or surgery. Herniations of temporal lobe parenchyma into arachnoid pits/cephaloceles along the sphenoid wing may be responsible for an epileptogenic focus (Figure 25) [97]. Multiplanar CT reformats are useful to demonstrate the location and size of bony defects, while MRI is better suited to characterize the contents of a cephalocele. MRI shows high signal on T2WI due to the presence of CSF, as well as varying degrees of gliosis in herniated brain tissue, and coronal thin slice 3D T1 and CISS sequences are particularly helpful (Figure 26) [95]. MRI is also useful in the determination of possible associated anomalies in patients with encephaloceles, including an agenesis of the corpus callosum and holoprosencephaly, and provides valuable information for surgical planning [2,39].

**Figure 27:**
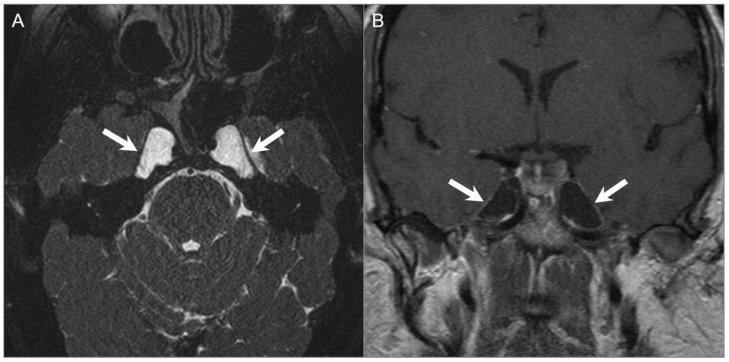
Meckel cave/petrous apex cephaloceles. (**A**) Axial CISS and (**B**) postcontrast coronal T1W images show well-defined expansion of both Meckel caves with CSF signal intensity extending to the anterior margins of the petrous apices (arrows).

Meckel cave cephaloceles can be congenital or acquired and may herniate into the petrous apex. They are believed to be caused by either normal CSF pulsation or chronically increased intracranial pressure transmitted into the Meckel cave through a patent porus trigeminus (Figure 27) [98,99]. Meckel cave cephaloceles in patients with idiopathic intracranial hypertension are often associated with an empty sella, are usually bilateral, and occur more often in women than men [2,100]. Their CT and MRI features are the same as other cephaloceles. In most cases, they are incidental and regarded as “do not touch” lesions, although they may be rarely associated with trigeminal neuralgia, neuropathy, headaches, CSF otorrhea, or hearing loss [2,99].

### 5.5. Sinonasal Mucocele

Sinonasal mucoceles develop due to the continuous accumulation of secretions in a paranasal sinus or nasal cavity with obstructed drainage. Mucoceles are associated with bony expansion and sinus wall remodeling, with the frontal sinuses being most commonly affected, followed by the ethmoid sinuses. Enlarging mucoceles may distort the local anatomy and exert mass on adjacent structures, typically affecting the orbit and extending intracranially in severe cases. Associated infection can lead to subdural empyema, meningitis, cerebral abscess, orbital subperiosteal abscess, and subcutaneous Pott’s puffy tumor [2,101].

**Figure 28:**
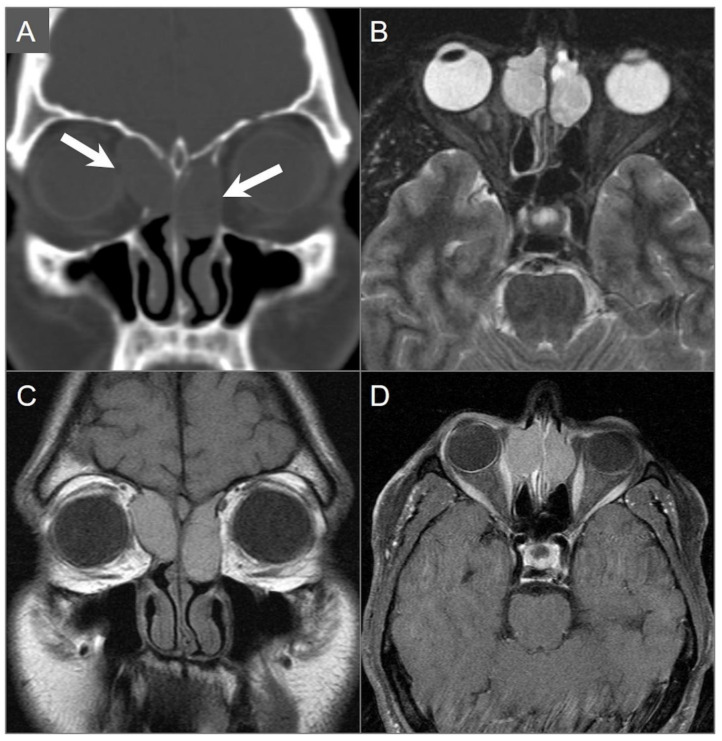
Mucocele. (**A**) Coronal CT image shows well-defined hypodense lesions in the bilateral ethmoid air cells with bony scalloping and dehiscence of the bilateral lamina papyracea. Additionally, note the extension into the orbits, which are greater on the right side (arrows). (**B**) Axial fat-suppressed T2W image shows moderate hyperintense signal in the lesions. (**C**) Coronal noncontrast T1W image demonstrates moderate corresponding hyperintense signal consistent with proteinaceous contents. (**D**) Axial postcontrast fat-suppressed T1W image demonstrates thin peripheral enhancement consistent with compressed mucosa. Mild mass effect on the right medial rectus muscle is noted.

On CT, the complete opacification of the affected sinus is typical, along with thin peripheral enhancement, bone resorption, and thinned sinonasal walls and septations. In some cases, areas of complete bone resorption may be present, leading to dehiscence and extension of the mucocele into adjacent tissues [101,102]. On MRI, the signal intensity of mucoceles differs depending on its content, with high water content appearing hyperintense on T2WI and high protein content appearing hyperintense on T1WI. Thin discrete rim enhancement on contrasted T1WI images usually represents compressed sinus mucosa (Figure 28) [2,39,102].

### 5.6. Invasive Fungal Sinusitis

Invasive fungal sinusitis is the most aggressive form of fungal sinusitis and a source of significant morbidity and mortality. It is seen mainly in immunocompromised patients such as those with diabetes mellitus, neutropenia, and advanced AIDS. Patients usually present with dramatic symptoms, including a rapid development of fever, nasal congestion, epistaxis, facial pain, and a loss of sensation in the malar tissues. The disease progresses rapidly and is associated with high mortality (50–80%), mainly due to vascular invasion and systemic dissemination. Extension into the nasopharynx, orbit, cavernous sinus, or intracranial compartment is frequent, resulting in the loss of vision, proptosis, and neurological deficits [103,104].

**Figure 29:**
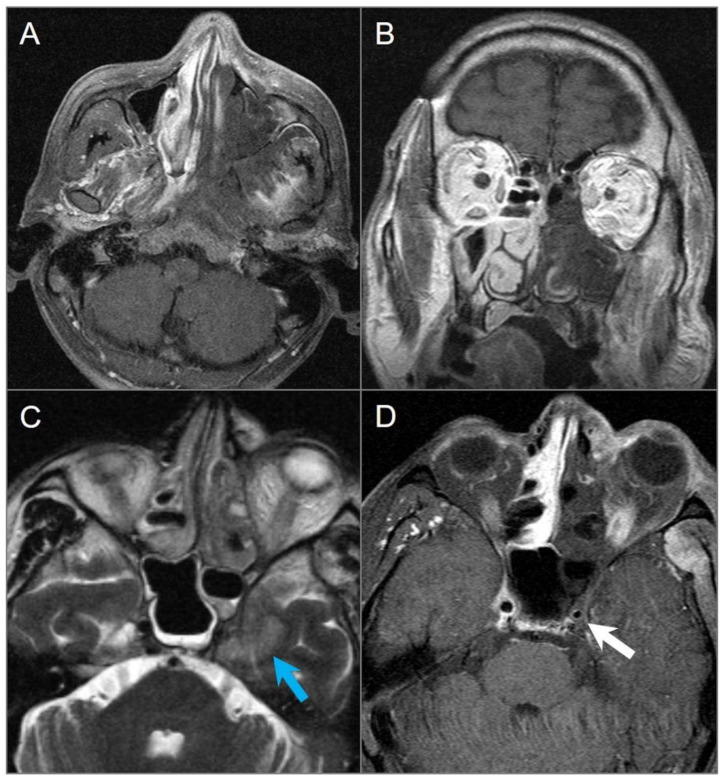
Invasive fungal sinusitis in a 20-year-old male patient. (**A**) Axial and (**B**) coronal postcontrast T1W images show large areas of hypointense non-enhancing soft tissue involving the left nasal cavity, maxillary sinus, nasal turbinates, masticator space, and nasopharynx, consistent with devitalized tissue (‘black turbinate’ sign). (**C**) Axial T2W and (**D**) postcontrast fat-suppressed T1W images reveal extension into the left cavernous sinus with left cavernous ICA encasement and narrowing (white arrow). There is also parenchymal edema in the left anteromedial temporal lobe (blue arrow).

Imaging manifestations can range from subtle findings in the early phase to a very aggressive disease that may mimic a malignant tumor. CT may reveal mucosal thickening with variable opacification of the affected sinuses, a prominence of the nasal mucosa, and fat stranding in the retroantral region, orbit, masticator space, and pterygopalatine fossa. Bone destruction is usually present to varying degrees, but in some cases, the bone may appear intact. Notably, the absence of bone destruction does not exclude spread to surrounding soft tissues, as Aspergillus tends to invade blood vessels even through intact bone [2]. On MRI, the T1 and T2 signal in the affected sinuses depends on the presence of water, protein, and fungal material. Fungal elements typically cause very low T2WI signal similar to an air signal void, due to an accumulation of metals such as iron and manganese. The surrounding soft tissues may be involved with varying degrees of inflammation and edema, appearing hypointense on T1W and hyperintense on T2W sequences (Figure 29) [2,39]. Notably, enhancement may be absent in areas of devitalized tissue, both in the sinonasal mucosa (the ‘black turbinate’ sign) and surrounding soft tissues. In such cases, vascular involvement is frequently observed, and the use of high-resolution vessel wall MRI is valuable in characterizing vasculitis by demonstrating an enhancement of the vascular wall in the areas of inflammation [105].

### 5.7. Petrous Apex Trapped Fluid

The petrous apex is susceptible to pathologic processes that affect the middle ear and mastoid air cells, such as inflammation, infection, and obstruction. These conditions may lead to the accumulation of fluid in the petrous apex, which can persist even after the inflammatory process and clinical symptoms have resolved. Trapped effusion is a common incidental finding on MRI, which can result in an asymmetric appearance of the petrous apex with variable T1 and T2 signal, depending on the protein content [2,71]. Signal intensity usually follows that of simple fluid on T1WI and T2WI, but in rare cases, intermediate or hyperintense T1 signal can be confused with other conditions, including cholesterol granuloma, mucocele, enhancing neoplasm or petrous apicitis. Noncontrast T1WI should be evaluated to ensure that one is looking at intrinsic signal hyperintensity rather than true enhancement. In equivocal cases, CT can be a complementary tool and will demonstrate opacification of the petrous apex with preserved septations and no evidence of bony expansion, cortical disruption, or trabecular erosion [71].

### 5.8. Petrous Apicitis

**Figure 30:**
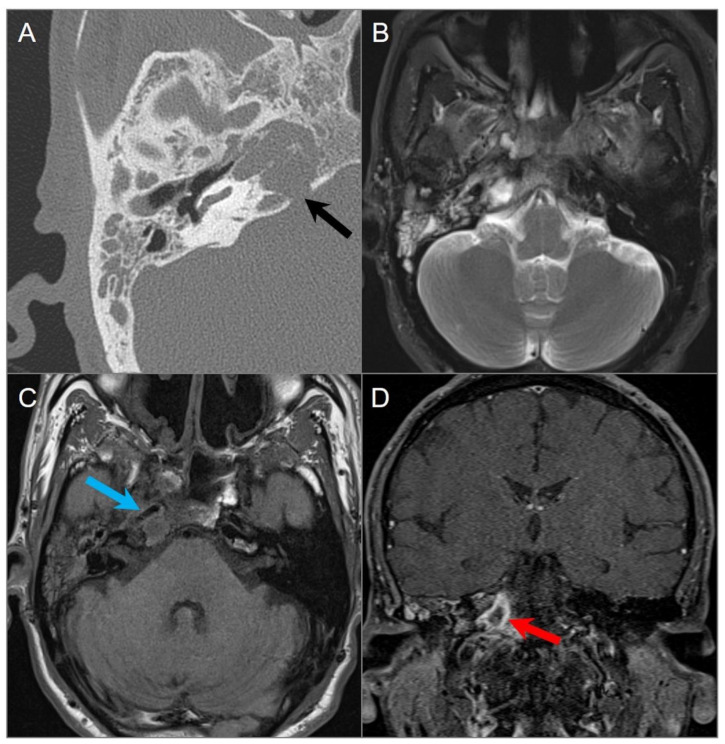
Petrous apicitis. (**A**) Temporal bone CT image shows opacification of the right mastoid air cells and external auditory canal with thickening of the tympanic membrane consistent with external otitis and mastoiditis. There is opacification of the right petrous apex with dehiscence of its posteromedial margin (black arrow). (**B**) Axial T2W and (**C**) noncontrast T1W images demonstrate fluid in the mastoid air cells, middle ear cavity, and petrous apex with associated thickening of the carotid artery wall, resulting in mild luminal narrowing (blue arrow). (**D**) Coronal postcontrast fat-suppressed T1W image reveals a peripherally enhancing collection within the right petrous apex consistent with abscess (red arrow).

Petrous apicitis is a rare complication of otomastoiditis, which occurs when infection spreads from the middle ear to the petrous apex via pneumatized air cells. Progression of purulent exudate leads to the destruction of air cell epithelium and demineralization of bony trabeculae. The infection can then spread to the bone marrow, causing a localized skull base osteomyelitis. The involvement of adjacent structures such as the carotid canal can result in vasospasm or arteritis of the internal carotid artery. Additionally, an involvement of emissary veins can cause thrombophlebitis and sinus thrombosis, and the infection can extend intracranially, resulting in meningitis, encephalitis, and intracranial abscess. CT can show opacification of preexisting apical air cells with associated otomastoiditis, mild expansion of the petrous apex, and permeative osseous destruction. However, bone changes may be subtle or absent early in the infectious process despite severe symptoms. On contrast-enhanced CT, petrous apicitis may appear as a heterogeneous enhancing mass that mimics a neoplasm. MRI is complementary to CT in the diagnosis of petrous apicitis and determination of the disease extent (Figure 30) [2,71]. DWI is a valuable tool in the diagnosis of petrous apicitis when used in conjunction with other conventional MRI findings. It can reveal restricted diffusion in the petrous apex caused by purulent material. However, it is crucial to differentiate these findings from other petrous apex lesions that also exhibit restricted diffusion, such as cholesteatoma and highly cellular neoplasms. This can be usually achieved by analyzing the findings of other conventional MRI sequences alongside DWI.

### 5.9. Paget Disease

Paget disease of the bone is a chronic non-inflammatory disease characterized by localized excessive bone remodeling that affects widespread, non-contiguous areas of the skeleton. It is the second most common bone disease after osteoporosis, occurring in approximately 3–4% of the population over the age of 50, with a slight male predominance [106,107]. Although most patients are asymptomatic, pagetic bone can cause clinical manifestations, such as calvarial enlargement, bone pain, local tenderness, headache, CN compression, and foraminal stenosis. Sensorineural and conductive hearing loss may occur due to a compression of CN VIII or due to loss of bone mineral density of the otic capsule and a fixation of the middle ear ossicles, respectively. In rare cases, osteosarcoma may arise in the affected bone, occurring in about 0.3% of patients [106].

**Figure 31:**
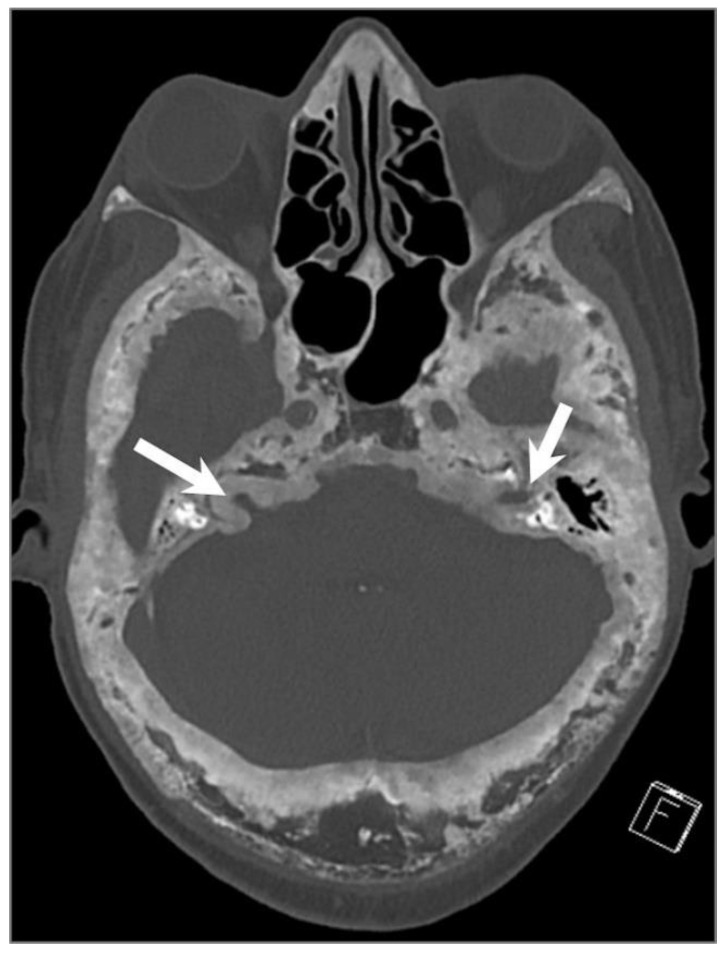
Paget’s disease. Axial CT image shows characteristic mixed lytic and sclerotic areas (‘cotton wool’ appearance) with thickened trabeculae, bone expansion, cortical thickening, and deformity of the calvarium and skull base. There is narrowing of the bilateral internal auditory canals due to cortical thickening (arrows).

Paget disease of the bone is diagnosed primarily by radiological examination, which shows three distinct stages: lytic, mixed, and sclerotic. In the early stages, lytic activity predominates, causing focal osteolytic lesions. As the disease progresses, sclerosis becomes the predominant feature, leading to the characteristic cotton wool appearance with mixed lytic and sclerotic areas, along with thickened trabeculae, bone expansion, cortical thickening, and osseous deformity (Figure 31) [106,107,108]. Radiological features are usually distinctive, but occasionally, the differential diagnosis of osteoblastic metastases needs to be considered, although the latter are not associated with trabecular or cortical thickening [108].

### 5.10. Ecchordosis Physaliphora

**Figure 32:**
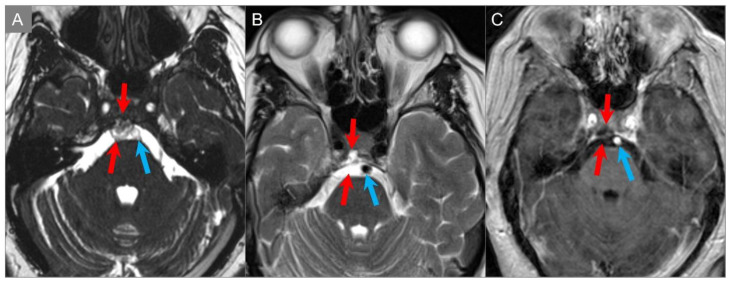
Ecchordosis physaliphora. (**A**) Axial CISS, (**B**) axial T2W, and (**C**) axial postcontrast T1W images reveal a small, lobulated, and markedly T2 hyperintense lesion in the clivus. There is no associated contrast enhancement. The lesion protrudes into the prepontine cistern (red arrows) abutting the ventral pons and basilar artery (blue arrows).

Ecchordosis physaliphora is a rare retroclival notochordal remnant that is usually asymptomatic and found in approximately 2% of autopsies. It is most commonly located in the retroclival prepontine region, but can also be seen anywhere along the midline from the skull base to the sacrum [2,74]. On high-resolution CT, it appears as a well-demarcated and smoothly corticated bony clival defect without aggressive features. Occasionally, a pathognomonic stalk-like connection with the clivus can be demonstrated on high resolution CT and MRI, particularly on CISS sequences. Ecchordosis physaliphora can be distinguished from its pathologic counterpart chordoma based on its imaging presentation, which includes a size smaller than 6 mm, the presence of CSF attenuation, and the absence of contrast enhancement or osseous destruction (Figure 32) [2,109].

### 5.11. Giant Aneurysm

**Figure 33:**
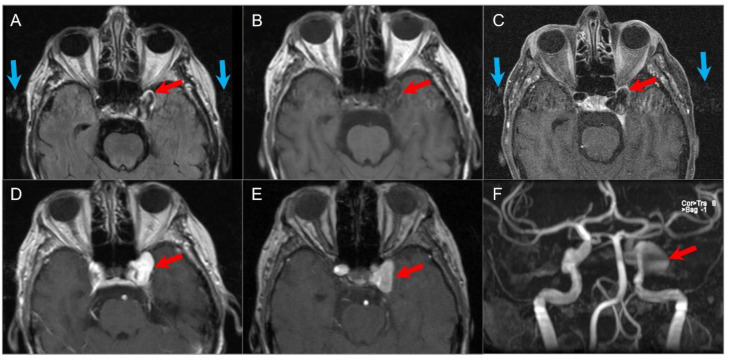
Giant left cavernous ICA aneurysm. (**A**) Axial FLAIR, (**B**) T1W, and (**C**) black-blood T1W vessel wall imaging show a giant left cavernous ICA aneurysm (red arrows) with pulsation artifact along the phase-encoding direction (blue arrows) and internal heterogeneous signal on FLAIR. (**D**) Axial postcontrast T1W image reveals homogenous enhancement of the aneurysm. (**E**) TOF MR angiography source and (**F**) MIP images show bright flow-related signal in the aneurysm.

Giant intracranial aneurysms, defined as larger than 25 mm in diameter, appear as expansile lesions of the cavernous sinus. They usually arise from the cavernous or supraclinoid internal carotid artery and can cause significant skull base erosion, extending into the paranasal sinuses, parapharyngeal space, and infratemporal fossa. Their recognition is critical due to treatment implications and, most importantly, to avoid a catastrophic biopsy. The imaging appearance of giant aneurysms depends on whether they are non-thrombosed or partially or completely thrombosed. Patent areas of an aneurysm will typically present with a prominent signal void on MRI due to rapid blood flow. Signal intensity is heterogeneous in the thrombosed portions of an aneurysm, which reflects the presence of different stage blood products and sometimes calcifications. The presence of flow-related artifacts in the phase-encoding direction may be helpful in the diagnosis (Figure 33). A potential pitfall on noncontrast CT is mistaking a largely patent aneurysm for a cavernous sinus meningioma when a CTA is not performed. CTA and MRA are useful to confirm the diagnosis and delineate the aneurysm [6,110,111].

## 6. Conclusions

The skull base is a complex and challenging anatomical region due to the presence of crucial neurovascular structures that enter and exit the cranial vault. Owing to its deep location, it is not amenable to direct clinical examination aside from surgical exploration. There are various benign and malignant entities as well as tumor mimics that may arise from the skull base and surrounding structures, which pose diagnostic and therapeutic challenges. Radiologic imaging is the cornerstone for detection, differential diagnosis, treatment planning, and follow-up of skull base tumors. CT and MRI are the primary imaging modalities and complement each other in narrowing the differential diagnosis and planning treatment. Given the diverse nature of tumors and mimics that affect the skull base, some of which share overlapping clinical and imaging manifestations, a thorough analysis of relevant neuroimaging features is important for accurate lesion characterization.

## Data Availability

Not applicable.
